# Transcriptome responses of *Streptococcus mutans* to peroxide stress: identification of novel antioxidant pathways regulated by Spx

**DOI:** 10.1038/s41598-017-16367-5

**Published:** 2017-11-22

**Authors:** Jessica K. Kajfasz, Tridib Ganguly, Emily L. Hardin, Jacqueline Abranches, José A. Lemos

**Affiliations:** Department of Oral Biology, University of Florida College of Dentistry, Gainesville, FL 32608 USA

## Abstract

The oxidative stress regulator Spx is ubiquitously found among Gram-positive bacteria. Previously, we reported identification of two Spx proteins in *Streptococcus mutans* – SpxA1 was the primary activator of oxidative stress genes whereas SpxA2 served a backup role. Here, we used RNA sequencing to uncover the scope of the H_2_O_2_ (peroxide)-stress regulon and to further explore the significance of Spx regulation in *S. mutans*. The transcriptome data confirmed the relationship between Spx and genes typically associated with oxidative stress, but also identified novel genes and metabolic pathways controlled by Spx during peroxide stress. While individual inactivation of newly identified peroxide stress genes had modest or no obvious consequences to bacterial survival, a phenotype enhancement screen using the ∆*spxA1* strain as background for creation of double mutants revealed that four of the five genes inactivated were required for stress survival. Physiological and biochemical assays validated, at least in part, the transcriptome data indicating that SpxA1 coordinates transcriptional changes during peroxide stress that modify global metabolism and facilitate production of antioxidants. Collectively, our findings unraveled the scope of the peroxide stress regulon and expand the repertoire of oxidative stress genes in *S. mutans*, shedding new light on the role of Spx regulation.

## Introduction

*Streptococcus mutans* is considered a major etiologic agent of dental caries due to three main attributes: (i) a capacity to form biofilms on tooth surfaces (dental plaque), (ii) an ability to convert dietary carbohydrates to lesion-inducing lactic acid, and (iii) an ability to adapt to sudden environmental changes in dental plaque^1^. To thrive at low-pH values, *S. mutans* activates the acid tolerance response (ATR), a genetic and physiologic adaptive mechanism that is relatively well-understood^1,2^. The ATR is accomplished by upregulation of the membrane-associated F-ATPase, induction of pathways that contribute to cytoplasm buffering and changes in membrane fatty acid composition, among other processes^1,2^.

While the ATR has been studied in some detail, oxygen metabolism and the mechanisms utilized by *S. mutans* to cope with oxidative stress have received limited attention. The initial notion that the dental plaque (biofilm) environment was virtually anaerobic has been now replaced by evidence that the oral microbial community as a whole has a high capacity to reduce oxygen, resulting in the generation of a variety of toxic reactive oxygen species (ROS) such as H_2_O_2_ and superoxide^3^. For example, members of the mitis group of streptococci (e.g. *S. gordonii* and *S. sanguinis*), which cohabit the dental biofilm with *S. mutans*, are net producers of H_2_O_2_. It follows that an inverse correlation between proportions of *S. mutans* and mitis streptococci has been observed in health and disease, with high numbers of *S. mutans* associated with caries and high proportions of mitis streptococci associated with oral health^4,5^. In addition, H_2_O_2_ present in oral hygiene and tooth bleaching products may represent another source of peroxide stress for oral bacteria^3^. Ultimately, via free radical formation as a result of the Fenton reaction in the presence of iron, the presence of high levels of H_2_O_2_ can rapidly cause irreversible cellular damage by attacking membrane lipids, triggering mismetallation of enzymes, directly damaging proteins through oxidation of sulfurous amino acids and metal-binding sites, and by disturbing DNA integrity^6^.

Global transcriptional studies following exposure to H_2_O_2_ have been extensively used to obtain new insights into the peroxide stress response mechanisms of bacteria. In *Escherichia coli*, the peroxide stress response is largely controlled by the OxyR regulator that activates transcription of reactive oxygen species (ROS) scavenging, iron homeostasis and disulfide reduction systems^7^. In the soil organism *Bacillus subtilis*, peroxide stress responses are governed by σ^B^ and the thiol-sensing OhrR, as well as the peroxide-sensing PerR, which activate (σ^B^ and OhrR) or derepress (PerR) transcription of systems involved in ROS scavenging, iron homeostasis, DNA repair and manganese uptake^8^. In *Staphylococcus aureus*, microarray analysis has linked DNA repair pathways, iron uptake and storage, and anaerobic metabolism to peroxide stress^9^. Moreover, Spx is another major transcriptional regulator of Gram-positive bacteria involved in oxidative stress responses, principally by activating transcription of genes involved in thiol homeostasis and detoxification^10–14^. While the bulk of the work that has contributed to a better understanding of Spx function was conducted with the Gram-positive paradigm *B. subtilis*, evidence is now accumulating that Spx proteins have similar regulatory functions in many other bacteria and are critical for virulence of several Gram-positive pathogens^10,12,14–17^.

Previously, we reported the identification of two Spx proteins in *S. mutans* that we initially named SpxA and SpxB but have recently renamed SpxA1 and SpxA2^12,13,18^ to avoid confusion with the streptococcal pyruvate oxidase SpxB^19^. Deletion of the *S. mutans spxA1* resulted in increased sensitivity to oxidative stresses, a phenotype that was significantly enhanced in the double Δ*spxA1/*Δ*spxA2* strain^12^. Transcriptional profiling of the Δ*spx* strains and *in vitro* transcription assays confirmed that SpxA1 plays a primary role in directly activating transcription of well-known oxidative stress genes such as *ahpC* (alkyl hydroperoxidase), *dpr* (iron-binding protein) and *sodA* (superoxide dismutase)^11–13,20^. SpxA2, however, appears to serve as a backup for SpxA1 in the activation of oxidative stress genes while its primary function may be to control transcription of genes involved in cell envelope homeostasis^12^.

In this study, we used RNA deep sequencing (RNA-Seq) to identify changes in the transcriptome of *S. mutans* after a brief exposure to H_2_O_2_ and used functional genomics and physiological approaches to characterize pathways newly associated with peroxide stress survival. In its totality, the present study unraveled the scope of the peroxide stress regulon of *S. mutans* and identified several new Spx-regulated pathways that are important for peroxide survival.

## Results

### Overview of the H_2_O_2_ stress transcriptome of *S. mutans* UA159

Previously, we used microarrays to compare the transcriptome of *S. mutans* UA159 and ∆*spx* strains, though that study was performed in the absence of any stress^12^. In addition, we have used quantitative real-time PCR (qRT-PCR) to examine the transcriptional profile of a selected number of Spx-regulated genes in response to H_2_O_2_ stress^11,13^. In these investigations, we found that exposure of mid-log phase cultures to 0.5 mM H_2_O_2_ for 5 min was optimal for induction of known oxidative stress genes such as *ahpCF*, *dpr* and *sodA* ^11,13^. Here, we used the same parameter to uncover the peroxide stress regulon of *S. mutans* UA159 via RNA-Seq. As compared to cells grown in the absence of stress (control), approximately 7% of the *S. mutans* UA159 genome showed altered transcription with 100 genes upregulated and 39 genes downregulated after H_2_O_2_ stress (Table S1; *P* ≤ 0.05). The differently expressed genes were grouped into 8 functional categories (Fig. S1) with genes encoding amino acid biosynthesis, DNA metabolism, and hypothetical proteins highly represented in the list of upregulated genes whereas genes coding for hypothetical proteins accounted for more than 50% of the downregulated genes followed by genes involved in carbon metabolism (~20% of the total number of downregulated genes). A subset of the differentially expressed genes was selected and used for quantitative real-time (qRT) PCR analysis for validation of the microarray data and the results were consistent with the expression trends observed in the RNASeq analysis (Tables S3 and S4).

As expected, peroxide stress resulted in the strong and rapid induction of ROS scavenging (*ahpCF*, *tpx*, and *sodA*) and thiol homeostasis (*gor*, *gst*, *trxA* and *trxB*) genes (Table 1). Recently, we also performed a study focusing on previously uncharacterized genes that were positively regulated by SpxA1^11^. Several of the genes from this previous study were also identified in the present RNA-Seq analysis, including *smu143* (putative transcriptional regulator), *smu144* (polypeptide deformylase), the *sufABCD* operon (Fe-S cluster assembly), *smu929* (conserved hypothetical protein) and *tehB* (tellurite resistance protein) (Table 1). While confirming previously reported trends^11–13,20,21^, the RNA-Seq analysis also uncovered new and interesting trends suggesting that peroxide stress triggered important metabolic shifts, often in an SpxA1-dependent manner, as detailed below.

**Table 1:** Expression changes of selected genes in *S. mutans* UA159, Δ*spxA1*, or Δ*spxA1*Δ*spxA2* following exposure to H_2_O_2_ stress.

Locus/gene name	Function	Fold change relative to:			
		WT H_2_O_2_ vs. WT control	Δ*spxA1* H_2_O_2_ vs. WT H_2_O_2_		Δ*spxA1*/*A2* H_2_O_2_ vs. WT H_2_O_2_
**Classic oxidative stress genes**					
SMU_0143c	polypeptide deformylase	3.2^a^	−3.6		−4.5
SMU_0144c	putative transcriptional regulator	2.8	−6.2		−7.3
SMU_0247	*sufC*, Fe-S cluster assembly	4.7	−4.2		−7.0
SMU_0248	*sufD*, Fe-S cluster assembly	4.7	−4.3		−7.6
SMU_0249	*sufS*, Fe-S cluster assembly	5.0	−4.4		−7.5
SMU_0250	*sufU*, Fe-S cluster assembly	4.4	−4.0		−7.0
SMU_0251	*sufB*, Fe-S cluster assembly	3.5	−4.2		−6.9
SMU_0463	*trxB*, thioredoxin reductase	3.8	−4.7		−6.4
SMU_0569	*feoA*, ferrous ion transport	ND^*b*^	6.1		5.8
SMU_0570	*feoB*, ferrous ion transport	−3.0	4.9		5.2
SMU_0571	*feoC*, ferrous ion transport	ND	4.7		5.5
SMU_0593	*furR*, Transcription	ND	−2.6		−3.9
SMU_0629	*sodA*, superoxide dismutase	5.1	−15.3		−50.8
SMU_0764	*ahpC*, alkyl hydroperoxide	11.5	−20.6		−74.8
SMU_0765	*ahpF*, alkyl hydroperoxide	10.5	−20.9		−70.2
SMU_0838	*gor*, glutathione reductase	5.9	−5.4		−11.2
SMU_0924	*tpx*, thiol peroxidase	7.3	−40.0		−120.4
SMU_0929	hypothetical protein	3.1	−13.9		−19.5
SMU_0995	*ftsA*, ferrichrome ABC transporter	ND	14.6		15.9
SMU_0996	*ftsB*, ferrichrome ABC transporter	ND	16.4		21.5
SMU_0997	*ftsC*, ferrichrome ABC transporter	ND	16.9		17.0
SMU_0998	*ftsD*, ferrichrome ABC transporter	ND	18.8		19.5
SMU_1117	*nox*, H_2_O-forming NADH oxidase	4.6	−19.9		−17.1
SMU_1296	*gst*, glutathione S-transferase	4.8	−5.3		−7.0
SMU_1297	3′-phosphoadenosine phosphatase	5	−8.6		−10.3
SMU_1645	*tehB*, tellurite resistance protein	2.6	−4.9		−9.5
SMU_1869	*trxA*, thioredoxin	4.8	ND		−23.5
**General stress and DNA repair genes**					
SMU_0188	*hsp33*, molecular chaperone	3.1	ND	ND	
SMU_0562	*clpE*, Clp protease, ATPase subunit	3.3	−2.6	−5.3	
SMU_0956	*clpL*, molecular chaperone	2.5	−1.9	ND	
SMU_1649	*smx*, exonuclease	3.1	−4.7	−12.4	
SMU_1650	*smn*, endonuclease III	ND	−3.6	−5.8	
SMU_1851	*uvrA*, UV repair excinuclease	4.0	ND	5.0	
SMU_1865	*mutY*, DNA glycosylase/lyase	3.1	−5.8	−9.6	
SMU_1954	*groEL*, molecular chaperone	2.6	ND	−3.7	
SMU_1955	*groES*, molecular chaperone	3.0	ND	−3.7	
SMU_2044	*relA*, (p)ppGpp synthetase/hydrolase	3.4	−3.4	−3.6	
**Energy-generating and histidine biosynthesis genes**					
SMU_0127	*adhA*, acetoin dehydrogenase	4.1	−11.2	−22.1	
SMU_0128	*adhB*, acetoin dehydrogenase	4.5	−12.1	−21.2	
SMU_0129	*adhC*, acetoin dehydrogenase	6.4	−11.5	−17.8	
SMU_0130	*adhD*, acetoin dehydrogenase	7.2	−10.6	−15.6	
SMU_0131	*lplA*, lipoate ligase	6.9	−10.6	−16.0	
SMU_0676	*gapN*	2.76	ND	ND	
SMU_1264	*hisF*, histidine biosynthesis	3.7	−2.8	−2.9	
SMU_1265	*hisA*, histidine biosynthesis	5.1	−2.9	−3.3	
SMU_1266	*hisH*, histidine biosynthesis	6.4	ND	−3.0	
SMU_1269	*serB*, histidine biosynthesis	4.9	ND	ND	
SMU_1270	*hisD*, histidine biosynthesis	5.8	−2.8	−3.0	
SMU_1272	*hisZ*, histidine biosynthesis	7.0	−3.1	−3.0	
SMU_1273	*hisC*, histidine biosynthesis	5.0	−2.6	−3.2	
SMU_1451	*aldB*, α-acetolactate synthase	3.6	−3.5	−4.9	
SMU_1452	*aldS*, α-acetolactate synthase	2.9	−3.6	−4.7	
SMU_1664	*acoB*, acetoin utilization	2.1	ND	−2.1	
SMU_1692	*pflA*, pyruvate formate lyase	3.6	−11.2	−14.0	
SMU_1867	*adhB*, alcohol dehydrogenase	3.7	ND	−21.5	

^a^All values shown were considered statistically significant. See Tables S1 and S2 for *p*-values. ^b^ND, no significant difference in gene expression was determined.

### Other stress genes affected by peroxide stress

In addition to previously identified Spx-regulated genes, a number of other stress survival genes were induced by the H_2_O_2_ treatment (Table 1). Considering that DNA damage is an immediate consequence of an oxidizing environment, it was not surprising to observe that the DNA repair genes, *exoA* (*smu1649)*, *uvrA* (*smu1851*) and *mutY* (*smu1865*), were induced by a minimum of 3-fold during peroxide stress. A number of general stress genes were also induced by peroxide stress, including the molecular chaperones *clpL*, *groES*-*EL* and *hsp33*, as well as the *clpE* ATPase. With the exception of *hsp33*, a redox-regulated chaperone that is activated by peroxide stress^22^, all other stress genes have been previously shown to be important for the acid stress survival of *S. mutans* ^18,23–25^ albeit their roles in peroxide survival have not been explored. Finally, the *relA* gene (*smu2084*, also known as *rsh* or *rel*) responsible for the production of the bifunctional (p)ppGpp synthetase/hydrolase was upregulated by 3.4-fold after peroxide stress. The RelA enzyme is responsible for activation of the stringent response, a conserved stress response mechanism to nutrient starvation^26^ that has been implicated in acid stress survival, biofilm formation and competence development of *S. mutans* ^27,28^. Notably, (p)ppGpp was previously shown to accumulate in *S. mutans* after H_2_O_2_ stress^29^.

### Metabolic re-routing as a consequence of peroxide stress

In addition to genes involved in oxidative and general stresses, our RNA-Seq analysis identified a number of genes involved in energy-generation (mainly pyruvate metabolism and fate) as upregulated after H_2_O_2_ treatment (Table 1). For example, transcription of genes of the *adhABCD* operon (*smu127*- *smu130*), predicted to encode the acetoin dehydrogenase (AoDH) complex, increased between 4.1 to 7.3 fold. Immediately downstream and apparently co-transcribed with the *adh* gene cluster is the *lplA* gene (*smu131*) encoding a lipoate ligase that may serve as a scavenger of lipoic acid from the environment. Of note, lipoic acid is an enzymatic co-factor of dehydrogenases, including the AoDH complex. Following the same trend of the *adh* genes, transcription of *lplA* was increased by 6.9-fold after the stress stimulus. Another operon that relates to acetoin metabolism (*smu1451-smu1452, aldB and alsS*) was upregulated by 3.6- (*aldB*) and 2.9-fold (*alsS*). The *alsS* gene encodes for an α-acetolactate synthase (ALS) that serves as a pyruvate sink consuming two molecules of pyruvate to yield one of acetoin. The acetoin may then be utilized by AoDH to produce acetaldehyde. In addition to acetoin/acetaldehyde metabolism, the 3.6-fold increased expression of pyruvate formate lyase (*pflA*, *smu1692*) was another indicator of a metabolic shift toward mixed fermentation, as this enzyme is involved in formation of formate from pyruvate. Collectively, these transcriptional changes suggest that peroxide stress triggers important changes in the fermentation profile of *S. mutans*.

### SpxA1 is the major transcriptional activator of the peroxide stress response

Above, we discussed the results of the transcriptional changes in the *S. mutans* UA159 (parent strain) transcriptome after H_2_O_2_ exposure. Here, we compare the transcriptional signatures of UA159 and *spx* mutant strains (∆*spxA1*, ∆*spxA2*, ∆*spxA1*∆*spxA2*) during peroxide stress (Table S2). A total of 230 genes were differentially expressed in ∆*spxA1* when compared to UA159, with 75 genes upregulated and 155 genes downregulated. Remarkably, the transcriptome of ∆*spxA2* after incubation with peroxide was nearly identical to that of the parent strain following stress, with only 14 genes upregulated and 5 genes downregulated. Despite the small number of differentially expressed genes in the ∆*spxA2* strain, the ∆*spxA1*∆*spxA2* double mutant strain showed an even greater number of differently expressed genes than the *∆spxA1* strain, with 170 upregulated genes and 264 downregulated. Among the 230 genes differently expressed in the *∆spxA1* strain when compared to UA159, 65% of these genes (n = 151) were also found in the comparison between the UA159 and ∆*spxA1*/∆*spxA2* strains (Fig. S2). In many cases, when genes were differentially expressed in both ∆*spxA1* and ∆*spxA1*∆*spxA2*, the difference in expression values as compared to UA159 were more extreme in the *∆spxA1∆spxA2* strain. This trend supports our earlier observations that SpxA2 serves a supporting or backup role for SpxA1^11–13^.

One difference that was noticed when comparing expression trends of the ∆*spx* mutants to the parent strain was the expression of the peroxide-inducible genes. In most cases, the archetypal oxidative stress genes were among the most strongly repressed genes in the ∆*spxA1* and ∆*spxA1*∆*spxA2* strains (Table 1). This was particularly noticeable for ROS scavenger genes such as *ahpCF*, *sodA* and *tpx*, which were downregulated by a minimum of 15-fold and as much as 120-fold in the ∆*spxA1* and ∆*spxA1*∆*spxA2* strains. This trend was also observed for genes involved in pyruvate metabolism (*adhABCD, aldB*, and *alsS*), DNA repair (*smx*, *uvrA* and *mutY*), histidine biosynthesis and general stress (*clpE*, *clpL* and *relA*) (Table 1). Further, transcription of two additional DNA repair genes (*smn* and *recA*) and of the iron-binding protein *dpr* was also significantly lower in the ∆*spxA1* and ∆*spxA1*∆*spxA2* strains when compared to the parent strain (Table 1). The operons encoding two major iron transporters*, feoABC* and *ftsABCD*, were strongly induced in ∆*spxA1* mutants. While the association of Spx as a repressor of the FeoABC system was previously reported^11^, the strong upregulation of the genes of the *ftsABCD* operon in the ∆*spxA1*∆*spxA2* strain (15 to 21-fold higher than the parent strain) confirms that SpxA1 regulates iron trafficking. Finally, transcription of the ferric uptake regulator *furR* (*smu593*), a well-characterized transcriptional repressor of iron transporters^30^, was downregulated in ∆*spxA1* and ∆*spxA1*∆*spxA2* suggesting that Spx may control iron uptake via the FurR regulator.

### A phenotype enhancement screen reveals hypersensitivity of double-mutant strains

**Figure 1:**
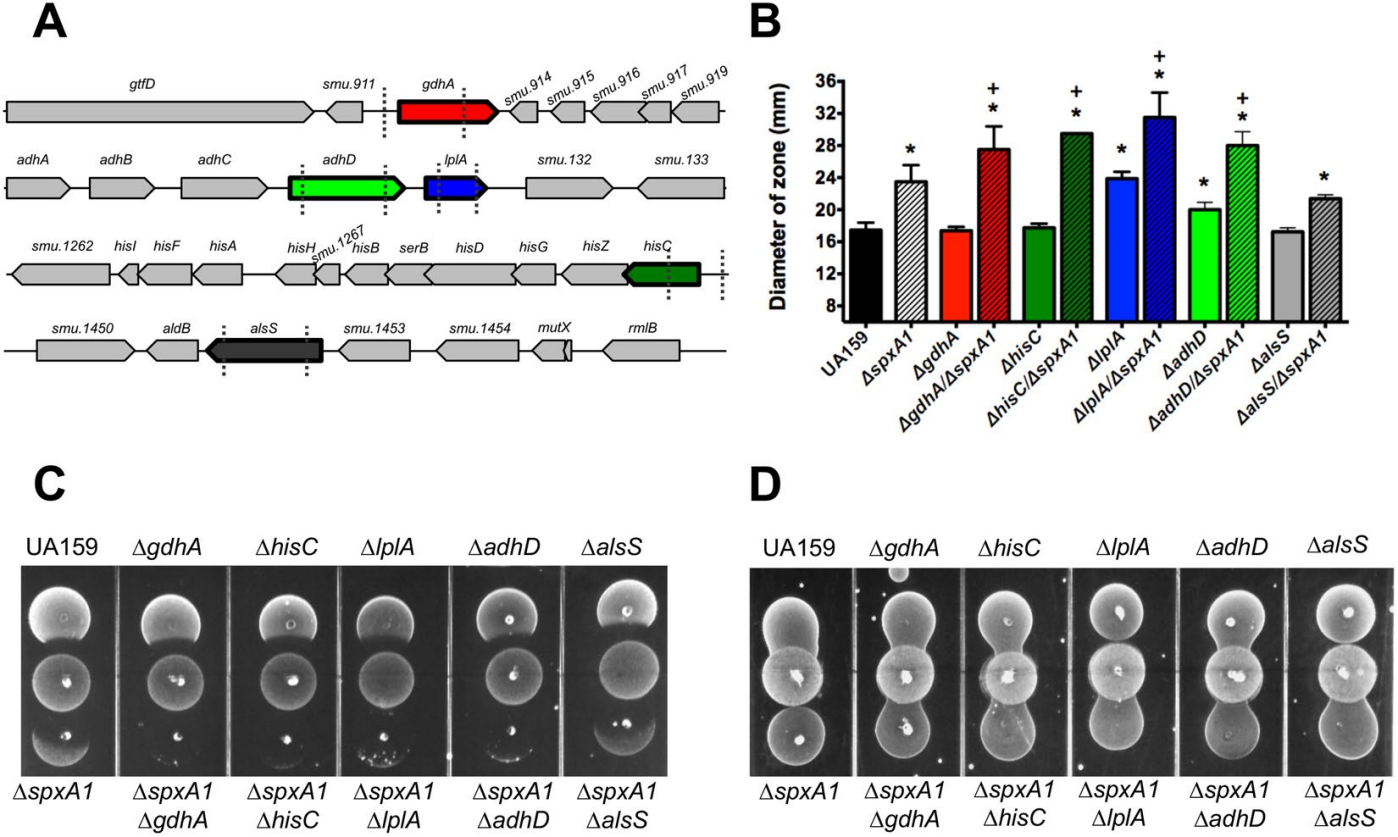
Phenotypic characterization of single and double (paired with ∆*spxA1*) mutant strains lacking *gdhA, adhD*, *lplA, hisC* and *alsS*. (**A**) Schematic representation of *gdhA*, *adhABCD*-*lplA*, *his* and *aldB*-*alsS* operons and flanking regions. Dashed lines indicate region of gene deletions. (**B**) H_2_O_2_ disc diffusion assay showing the diameters (in mm) of the zone of growth inhibition around discs saturated with 0.5% H_2_O_2_. (**C**) Growth inhibition of *S. mutans* UA159 and its derivatives by the peroxigenic *S. sanguinis* SK150 strain. (**D**) The *S. sanguinis*-*S. mutans* competition assay was repeated with catalase overlaid onto the *S. sanguinis* spot to inactivate H_2_O_2_. (*) compared to UA159, (+) - compared to ∆*spxA1*; *p* < 0.005 using Student’s *t*-test.

To further investigate the role of the SpxA1-regulated genes identified in the RNA-Seq analysis, we used a markerless strategy to delete the *adhD*, *alsS*, *hisC* and *lplA* genes, as this is the first time that these genes have been associated with SpxA1 (Fig. 1A). Using this approach, the ∆*adhD*, ∆*hisC* and ∆*lplA* strains were readily obtained. We also created a markerless deletion in *gdhA*, encoding a glutamate dehydrogenase responsible for the deamination of glutamate to KG. After several unsuccessful attempts to obtain a markerless *alsS* mutant, we used a non-polar kanamycin cassette to isolate the ∆*alsS* strain. All mutant strains were viable and, with exception of ∆*lplA* that showed a slightly longer lag phase, grew as well as the parent strain in the absence of stress (data not shown).

We then tested the ability of the mutants to tolerate peroxide stress. In a H_2_O_2_ disc diffusion assay, the ∆*adhD* and ∆*lplA* mutants were significantly more sensitive than the parent strain with the ∆*lplA* strain displaying a zone of inhibition comparable to the highly sensitive ∆*spxA1* strain (Fig. 1B). However, in a qualitative competition assay against a peroxigenic *S. sanguinis* strain, the differences between parent and all single mutant strains were subtle and unlikely to be biologically relevant (Fig. 1C). Nevertheless, it is not entirely surprising that single gene inactivation does not result in strong peroxide-sensitive phenotypes. While the physiological changes associated with those mutations may be part of the oxidative stress response of *S. mutans*, functional redundancy and genetic buffering may account for the lack of stronger phenotypes^31^. We and others have shown that genomic buffering could be overcome by introducing mutations of Spx-regulated genes into the ∆*spxA1* background strain^11,32^. We utilized this same approach here by introducing a marked *spxA1* deletion into the newly generated single mutants, and then compared the peroxide sensitivity of the double mutants to that of the ∆*spxA1* single mutant. This time, with exception of ∆*alsS*∆*spxA1*, all other double mutants (∆*adhD*∆*spxA1*, ∆*hisC*∆*spxA1*, ∆*lplA*∆*spxA1* and ∆*gdhA*∆*spxA1)* showed marked increases in peroxide sensitivity when compared to the single ∆*spxA1* mutant strain in both disc diffusion and *S. sanguinis* antagonism assays (Fig. 1B,C).

### The histidine biosynthesis operon is activated during peroxide stress

**Figure 2:**
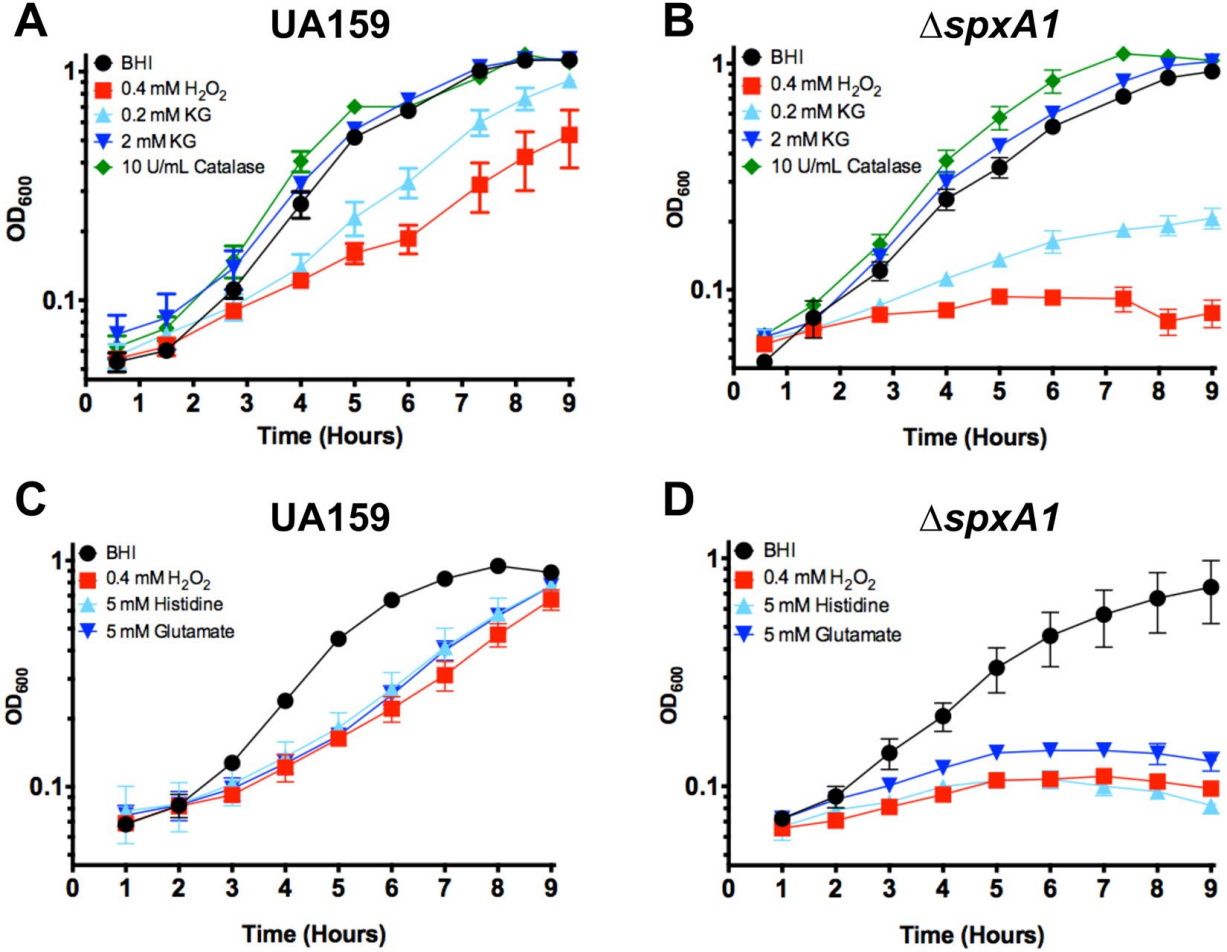
Addition of α-ketoglutarate (KG) abolishes *S. mutans* growth defect caused by H_2_O_2_ stress. Strains UA159 (**A**,**C**) or ∆*spxA1* (**B**,**D**) were grown in BHI broth in the absence or presence of 0.4 mM H_2_O_2_ with catalase (positive control), KG, histidine or glutamate added to the growth media using the concentration indicated in the figure.

It was also interesting to note that transcription of eight of the thirteen genes comprising the histidine biosynthesis (*his*) operon were upregulated (~3 to 7-fold) during peroxide stress (Table 1). In *Pseudomonas fluorescens*, histidine is earmarked for α-ketoglutarate (KG) production during oxidative stress^33^. A TCA cycle intermediate, KG is a potent antioxidant as it spontaneously reacts with H_2_O_2_ to generate succinate and CO_2_^34,35^. More specifically, some bacterial species are able to convert histidine to glutamate and ammonia in a multistep enzymatic reaction catalyzed by the genes of the *hut* (histidine utilization) operon^36^. Glutamate dehydrogenase is thereby provided with substrate to catalyze the deamination of glutamate to KG. To assess the possibility that production of KG via histidine-to-glutamate deamination could be part of the *S. mutans* oxidative stress response, we tested the effects of exogenously added histidine, glutamate or KG on growth of *S. mutans* in the presence of 0.4 mM H_2_O_2_. This concentration of H_2_O_2_ induced a prolonged lag phase and dramatically slowed growth of the UA159 parent strain (Fig. 2A) and completely abolished growth of the ∆*spxA1* strain (Fig. 2B). The addition of 0.2 mM KG to the growth media partially restored growth of both strains exposed to H_2_O_2_, whereas 2 mM KG completely reversed the growth defect of both strains (Fig. 2A and B). On the other hand, addition of histidine (Fig. 2C) or glutamate (Fig. 2D) (up to 5 mM) failed to rescue growth of the two strains.

**Figure 3:**
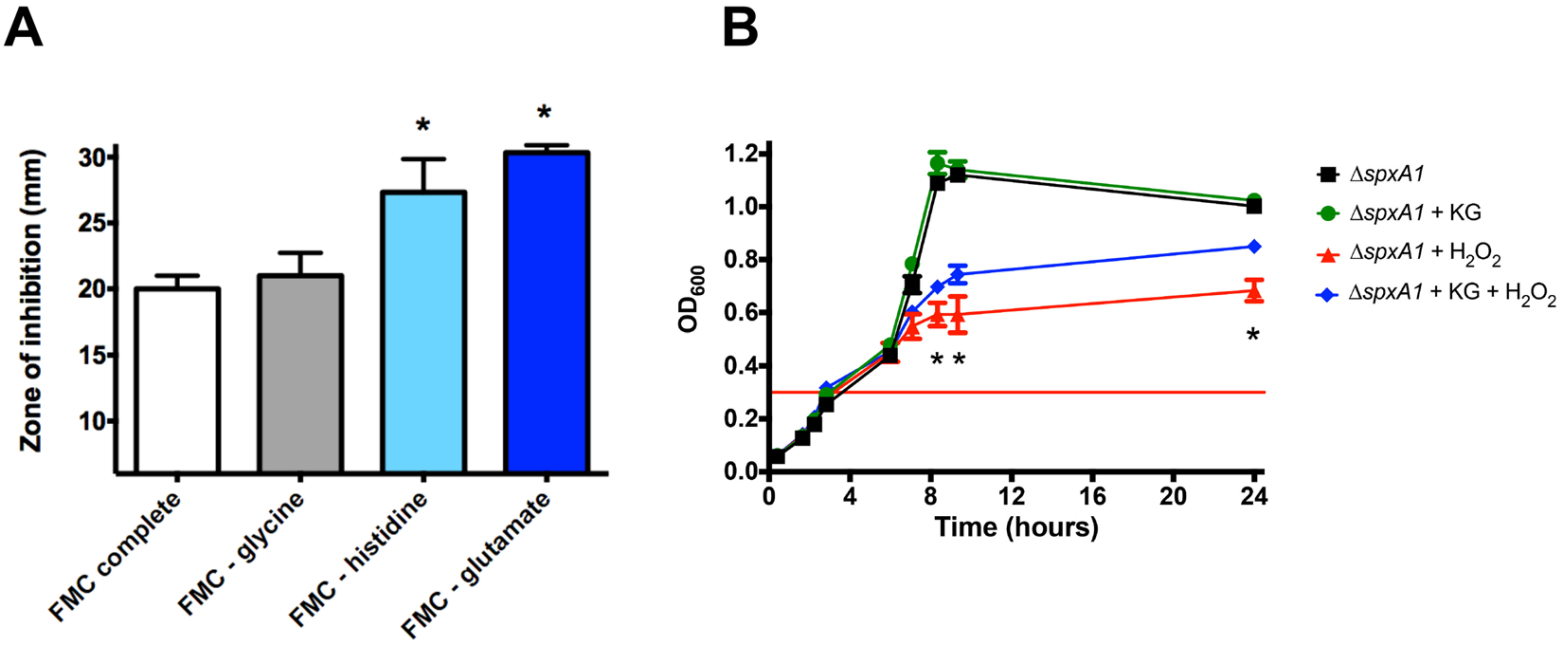
Physiological evidence that histidine, glutamate, and KG are associated with oxidative stress survival. (**A**) Depletion of histidine or glutamate increased sensitivity of *S. mutans* UA159 to H_2_O_2_. Sensitivity of *S. mutans* to 0.5% H_2_O_2_ delivered on paper discs was determined on agar plates composed of the chemically defined medium FMC, altered to omit histidine, glutamate, or glycine as indicated. The diameters of the zone of growth inhibition around the H_2_O_2_ discs were measured in mm. (*) indicates statistical significance as compared to FMC complete (white bar). (**B**) Pre-loading with KG offered protection from later exposure to H_2_O_2_. Growth of *S. mutans* ∆*spxA1* was initiated in the absence or presence of 2 mM KG. Upon reaching early log phase (OD_600_ = 0.3, red horizontal line), cultures were washed twice in phosphate buffered saline to remove extracellular KG. The cells were then resuspended in BHI in the absence or presence of 0.4 mM H_2_O_2_. (*) indicates statistical significance when comparing strains exposed to H_2_O_2_ in the presence or absence of KG (blue and red lines). (*) *p* < 0.01 using Student’s *t*-test.

Despite this initial evidence that KG protects *S. mutans* from peroxide stress, we cannot rule out that the spontaneous reaction between KG and H_2_O_2_ is occurring outside the cell since a similar level of protection was observed with catalase that is not expected to enter the cellular compartment (Fig. 2A and B). Thus, we also took a reductionist approach and used a disc inhibition assay to test whether histidine or glutamate deprivation would affect the peroxide tolerance of *S. mutans*. In this case, sensitivity to H_2_O_2_ was significantly increased when either histidine or glutamate were omitted from the growth media but not when glycine (randomly chosen as a control) was omitted (Fig. 3A). Of note, the omission of histidine, glutamate or glycine from the growth media did not affect growth rates of *S. mutans* (data not shown). As a further measure to address the concern that KG might be detoxifying only extracellular H_2_O_2_, a KG pre-loading experiment was performed. *S. mutans* cells were inoculated in the presence or absence of 2 mM KG. Upon reaching early log phase, the cultures were washed to remove any extracellular KG, then resuspended in medium containing H_2_O_2_. The H_2_O_2_-exposed cultures demonstrated a considerable defect in growth yield as compared to controls that had not been exposed to the stress (Fig. 3B). However, the cultures that had been provided with KG prior to the stress showed a significant recovery of final growth yield.

### The fermentative profile of peroxide-treated cells supports the altered transcriptome

**Figure 4:**
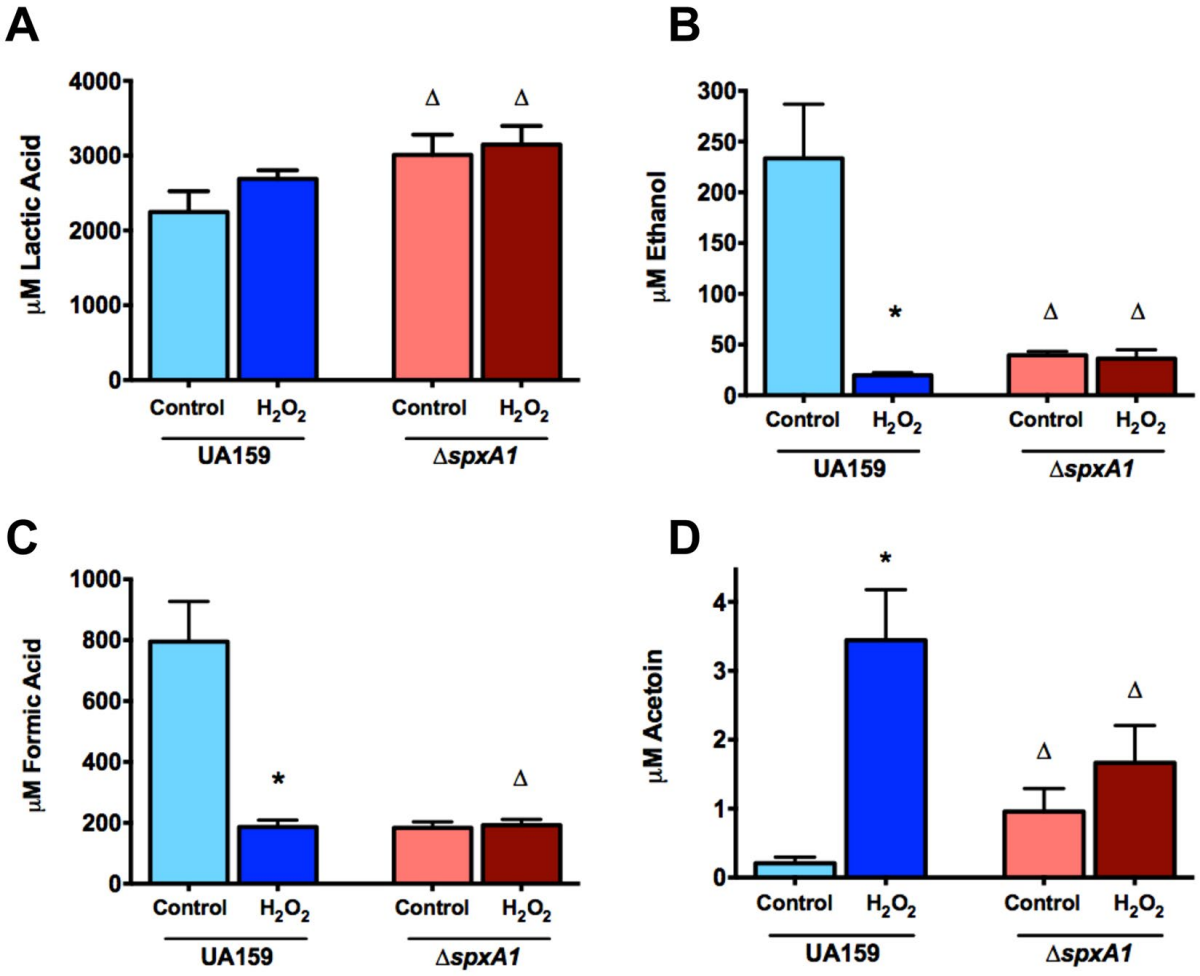
Metabolic profiles of stressed *S. mutans* UA159 and ∆*spxA1*. Cultures grown to early log phase (OD_600_ = 0.35) were exposed to 0.5 mM H_2_O_2_ for 60 minutes, while unstressed controls were incubated for the same period of time. Culture supernatants were harvested by centrifugation and used to determine the concentrations of lactic acid (**A**), ethanol (**B**), formic acid (**C**), and acetoin (**D**). (*) indicates statistical significance as compared to unstressed UA159. (∆) indicates statistical significance as compared to the equivalent UA159 culture. (*) or (∆) *p* < 0.02 using Student’s *t*-test.

As a preliminary step toward assessment of the impact of peroxide stress and SpxA1 on the fermentation profile of *S. mutans*, enzymatic assays were performed to measure metabolic end products stemming from the metabolism of pyruvate. Abundance of metabolites was measured from the culture supernatants of cells exposed to 0.5 mM H_2_O_2_ for 60 minutes, as compared to unstressed controls grown to the same optical density (OD_600_~0.6). Exposure to H_2_O_2_ resulted in minimal impact on lactic acid production by either the wild-type UA159 or ∆*spxA1* strains, albeit the ∆*spxA1* strain produced significantly more lactic acid (~25% more) than UA159 cultures (Fig. 4A). Interestingly, peroxide stress resulted in reduced ethanol production of approximately 10-fold in the parent strain as compared to unstressed UA159 cells, (Fig. 4B). On the other hand, the ∆*spxA1* strain failed to produce large quantities of ethanol regardless of the growth condition. Similar results were observed for formate that showed a 4-fold reduction after H_2_O_2_ stress in the parent strain but not in the ∆*spxA1* strain (Fig. 4C). In agreement with the H_2_O_2_-induced activation of the ALS genes (*alsS* and *aldB*) that convert pyruvate into the non-acidic end product acetoin, acetoin pools rose approximately 16-fold after peroxide treatment in the parent strain but not in the ∆*spxA1* strain (Fig. 4D). Because the *adhABCD* operon is predicted to convert acetoin into acetaldehyde and is also induced by peroxide stress, we also attempted to quantify acetaldehyde using a biochemical approach. However, we were unable to detect acetaldehyde above background levels under any growth condition, possibly due to its highly volatile nature.

## Discussion

In this report, we showed that in addition to the archetypal oxidative stress genes such as those involved in ROS scavenging and thiol homeostasis, Spx mediates transcription of genes involved in DNA repair, pyruvate metabolism and amino acid biosynthesis. To our knowledge, the study presented here is the first to uncover the peroxide transcriptome of a streptococcal species. Nonetheless, two microarray studies whereby *S. mutans* cells were exposed to oxidative stress by aeration^37^ or excess (8.4%) oxygen^20^ support our results as both studies reported increased expression of several genes identified in our study (*adhABCD, ahpCF*, *gor, lplA*, *sodA, tpx*, among others). It was also interesting to note the remarkable overlap between the peroxide transcriptome of the present study and the transcriptome signature of *S. mutans* during acid stress^38,39^. For example, progressive environmental acidification to pH 5.5 via glycolysis led to increased expression of the histidine operon, *adhABCD, aldB and alsS*, as well as several antioxidant (*ahpCF*, *sodA*, *tpx*), thiol homeostasis (*trxB*), DNA repair (*mutY* and *smn*) and general stress (*clpE*, *clpL* and *rel*) genes^38^. Similarly, a study evaluating the global transcriptional profile of *S. mutans* acid-shocked to pH 5 for two hours also described the induction of several peroxide-induced genes including *adhD*, *ahpCF, dpr* and *mutY* ^39^.

Previously, we showed that SpxA1 plays a major role in iron homeostasis by serving as a transcriptional activator of genes encoding for iron-binding protein (*dpr*), tellurite resistance (*tehB*), Fe-S cluster assembly (*sufABCD*) and peptide deformylase^11^ as well as a repressor of the ferrous iron transporter system (*feoABC*). If one takes into account that iron is a catalyst of the Fenton reaction, the involvement of Spx as a negative regulator of iron transport is logical. In fact, we have previously shown that the ∆*spxA1* strain is significantly more sensitive to the iron-dependent antibiotic streptonigrin^11^. Here, we expand the relationship between Spx and iron trafficking by confirming that transcription of *feoABC* was greater in the ∆*spxA1* and ∆*spxA1*∆*spxA2* strains (4 to 6-fold) but also by showing that the ferrichrome permease operon (*ftsABCD*) was strongly induced (15 to 21-fold) in the double mutant ∆*spxA1*∆*spxA2* strain. While iron is an essential micronutrient, the increased expression of iron transporters in ∆*spxA1* strains is likely to exacerbate the ROS stress imposed upon these strains that already have an impaired ability to activate antioxidant defenses and maintain iron homeostasis.

To begin to determine the significance of some of the genes identified in the RNASeq analysis in oxidative stress, we isolated and characterized isogenic deletion mutants lacking the *alsS* (∆*alsS*), *adhD* (∆*adhD*), *lplA* (∆*lplA*), *hisC* (∆*hisC*) and *gdhA* (∆*gdhA*) genes. While the ∆*adhD* and ∆*lplA* strains showed modest increases in sensitivity in H_2_O_2_ disc diffusion assays, our interpretation is that these single gene inactivations have little impact on the ability of *S. mutans* to cope with peroxide stress. It is not uncommon to observe a complete lack of detectable phenotypes from single gene deletions due to functional redundancy and, in this particular case, genetic interactions within the Spx regulon^40^. For example, when a single gene of the SpxA1 regulon is inactivated, a detectable phenotype may be masked by the robust response of other Spx-regulated genes. This “genetic buffering” phenomenon is evident in systematic gene deletion libraries whereby gene functions are rarely assigned based upon single gene deletions^40^. To overcome this limitation, we have used a “phenotype enhancement” approach^32^ whereby we paired each new mutation of an Spx-regulated gene with the *spxA1* deletion mutant creating a panel of double mutant strains^11^. By decreasing overall expression of SpxA1-regulated genes in the Δ*spxA1* strain background, we were able to unequivocally demonstrate that loss of *adhD*, *gdhA*, *hisC* and *lplA* markedly increased the peroxide stress sensitivity of the Δ*spxA1* strain.

Another interesting finding from the RNA-Seq analysis was that genes involved in pyruvate metabolism (*adhABCD*-*lplA*, *aldB, alsS*, and *pflA*) followed the same trend as the oxidative stress genes, e.g. induced by H_2_O_2_ stress in an Spx-dependent manner. The *lplA* gene encodes a lipoate ligase, the function of which may be to scavenge lipoic acid for use as an antioxidant and also to lipoylate enzymes that require this co-factor^41,42^. For example, lipoylation is known to be essential for the activity of dehydrogenases and the genetic proximity of *lplA* to the *adh* operon (AoDH) suggests that *lplA* may serve to provide this co-factor to AoDH. The protein products of the *aldB*-*alsS* operon are responsible for the conversion of pyruvate to acetoin, while genes of the *adhABCD* operon (acetoin dehydrogenase, AoDH) are predicted to catalyze the conversion of acetoin to acetaldehyde but may also work in reverse, augmenting acetoin production. To obtain the first glimpses into the metabolic profile of *S. mutans* under peroxide stress, we compared the production of lactate, ethanol, formate and acetoin in cells subjected to H_2_O_2_ stress. When compared to cultures grown in the absence of stress, the production of ethanol and formate was drastically reduced (10- and 4-fold, respectively) in the wild-type strain UA159 after peroxide stress whereas acetoin levels increased by 16-fold after stress. In addition, there was a small increase in lactate production after stress but the difference observed was not statistically significant. While the *pflA* gene, coding for the pyruvate formate lyase, is induced after H_2_O_2_ stress, the reduction in formate production after H_2_O_2_ stress is not unexpected given that the PflA enzyme is highly sensitive to oxidation^43^. The increased production in acetoin during peroxide stress is in line with the increased transcription of the ALS genes (*alsS* and *aldB*) that convert pyruvate into the non-acidic end product acetoin. Interestingly, acetoin pools have been shown to increase during aerobic growth in *S. mutans* ^44^. Moreover, acetoin can spontaneously react with oxygen and H_2_O_2_ in the presence of Fe^3+^ ^45^, even though it is not known if this reaction can occur *in vivo*. While these results indicate a linkage between acetoin production and oxidative stress that deserves further investigation, it should be noted that the very small amounts of acetoin produced (~0.01% of the total lactate produced under the same growth condition) may have little, if any, impact on cell physiology. Finally, ethanol, formate and acetoin pools remained largely unaltered in the ∆*spxA1* strain after H_2_O_2_ indicating that SpxA1 regulation is critical for the metabolic alterations observed during stress. Collectively, these results validate the transcriptome data and open new doors for more detailed metabolic studies.

In addition to the genes involved in pyruvate metabolism, transcription of eight of the thirteen genes comprising the histidine biosynthesis operon was induced by H_2_O_2_, also in an Spx-dependent manner. There are several, not mutually exclusive, explanations for the apparent increase in histidine biosynthesis during peroxide stress. First, histidine is particularly prone to metal-catalyzed oxidation that results in the formation of 2-oxo-histidine – a biological marker for assessing protein oxidation during stress^46^. Thus, it is possible that *S. mutans* increases histidine biosynthesis during oxidative stress to simply restore cellular histidine pools. Second, histidine has been proposed to function as a scavenger of hydroxyl radical and singlet oxygen, but not of H_2_O_2_ and superoxide anion^47^. In vertebrates, histidine-containing dipeptides such as carnosine protect neural cells by acting as antioxidants^48^. Finally, histidine can be enzymatically converted to glutamate, which in turn can be deaminated to form KG, a potent antioxidant that spontaneously reacts with H_2_O_2_ to generate succinate and CO_2_^33,34^. While *S. mutans* has an incomplete TCA cycle and cannot rely on this pathway for generation of KG, it can utilize the citrate metabolism pathway to generate KG from isocitrate via the aconitase enzyme (CitB). However, CitB is a cysteine-rich enzyme that becomes inactive during oxidative stress^49^, an indication that glutamate deamination may be the only source of KG for *S. mutans* during oxidative stress. Because histidine was shown to be earmarked for KG production in *P. fluorescens* during oxidative stress^33,35^, we tested the protective effects of KG, as well as histidine and glutamate, on growth and survival of *S. mutans* during oxidative stress. Our results clearly demonstrate that KG is a physiologically relevant antioxidant capable of attenuating the oxidative stress sensitivity of *S. mutans*. However, a homologous system to the histidine utilization (*hut*) operon responsible for the conversion of histidine to glutamate in some bacteria, was not identified in *S. mutans* ^36^. While *S. mutans* may use an alternative pathway to convert histidine into glutamate, the possible association of the histidine-to-glutamate-to-KG pathway in *S. mutans* oxidative stress response remains to be confirmed. Our future efforts will include direct quantifications of intracellular pools of amino acids and organic acids as well as the detection of 2-oxo-histidine levels.

**Figure 5:**
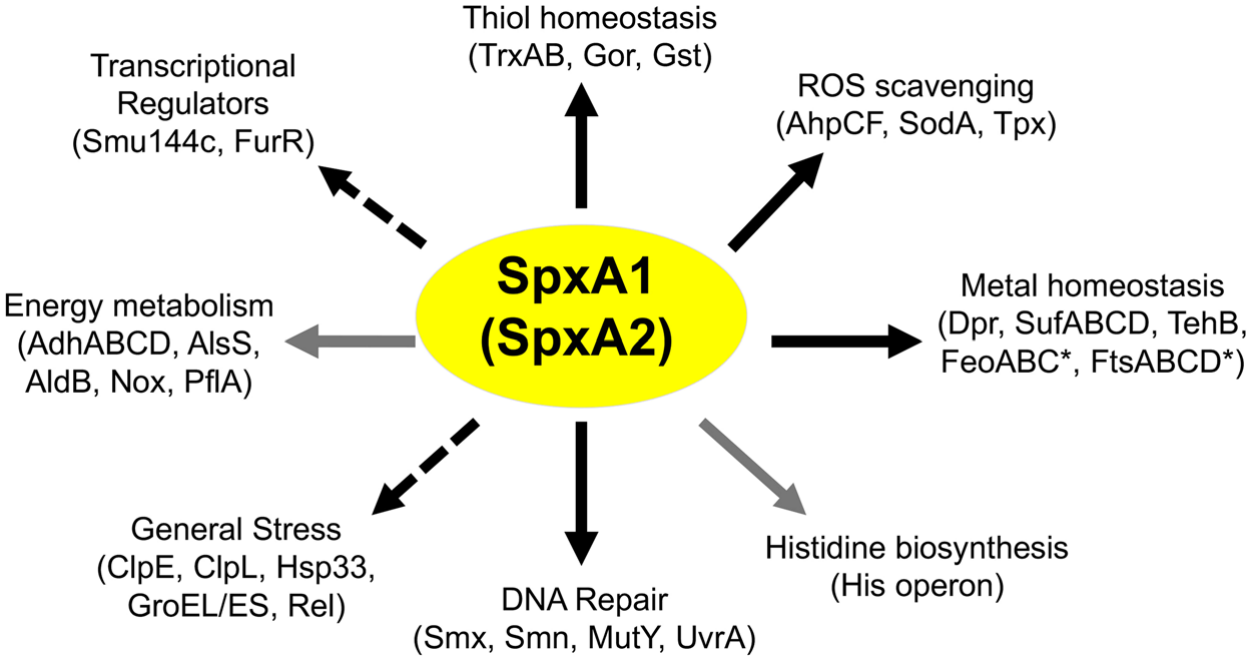
Antioxidant pathways of *S. mutans* regulated by Spx. Solid arrows indicate traits that have been validated through transcriptional and/or mutational analyses in the present (gray) or previous (black)^11–13^ studies. Dashed arrows denote pathways identified through transcriptome analysis that require further validation. (*) SpxA1 acts as a repressor of the *feoABC* and *ftsABCD* operons.

Based on the evidences provided in this study, we propose to expand the Spx-regulated pathways that are important for oxidative stress survival to include activation of DNA repair enzymes, histidine biosynthesis and alterations in pyruvate metabolism (Fig. 5). While it remains to be further dissected, our RNA-Seq analysis also indicates the existence of a crosstalk between the Spx regulators and classic stress survival systems, which may include activation of the stringent response and of stress chaperones. While other transcriptional regulators such as PerR and SloR participate in the oxidative stress responses of *S. mutans* ^50,51^, our collective results indicate that SpxA1, and to some extent SpxA2, function as the master regulators responsible for launching a rapid, multi-strategy defense towards oxidative insults in *S. mutans*. Because the Spx regulation is conserved among Gram-positive bacteria, the findings presented here are likely to have broader implications.

## Methods

### Bacterial strains and growth conditions for RNA-seq analysis

The bacterial strains used in this study are listed in Table 2. *S. mutans* UA159 and its derivatives were routinely grown in brain heart infusion (BHI) at 37 °C in a 5% CO_2_ atmosphere or, in the case of oxidative stress sensitive Δ*spx* strains, under anaerobic conditions (BBL Gaspack system, BD, Franklin Lakes, NJ). For RNA-Seq analysis, duplicate cultures were grown in BHI to an OD_600_ of 0.4, at which point control samples were harvested by centrifugation, while experimental samples were exposed to 0.5 mM H_2_O_2_ for 5 min before cell pellets were harvested by centrifugation and stored at −80 °C until use.

**Table 2:** Bacterial strains and plasmids used in this study.

Strains	Relevant genotype	Source
***Streptococcus mutans***		
UA159	Wild-type	Laboratory stock
∆*spxA1*	*spxA1*::Spec	18
∆*spxA2*	*spxA2*::Erm	18
∆*spxA1*∆*spxA2*	*spxA1*::Spec, *spxA2*::Erm	12
∆*gdhA*	markerless deletion of *gdhA*	This study
∆*gdhA*∆*spxA1*	*spxA1*::Spec, markerless *gdhA* deletion	This study
∆*lplA*	markerless deletion of *lplA*	This study
∆*lplA*∆*spxA1*	*spxA1*::Spec, markerless *lplA* deletion	This study
∆*hisC*	markerless deletion of *hisC*	This study
∆*hisC*∆*spxA1*	*spxA1*::Spec, markerless *hisC* deletion	This study
∆*adhD*	markerless *adhD* deletion	This study
∆*adhD*∆*spxA1*	*spxA1*::Spec, markerless *adhD* deletion	This study
∆*alsS*	*alsS*::Kan	This study
∆*alsS*∆*spxA1*	*spxA1*::Spec, *alsS*::Kan	This study
***Streptococcus sanguinis***		
SK150	Wild-type	Laboratory stock

### Construction of mutant strains

*S. mutans* strains bearing unmarked deletions in the *gdhA* (*smu913*), *lplA* (*smu131*), *hisC* (*smu1273*), or *adhD* (*smu130*) genes were created using natural genetic transformation techniques as described elsewhere^52^. Briefly, approximately 3-kb stretches of the DNA flanking the gene of interest at both the 5′ (primers 1 and 3) and 3′ (primers 4 and 5) ends were amplified using the primers listed in Table S5. For each gene, primers 3 and 4 encoded complementary sequences facilitating the annealing of the 5′ PCR product to the 3′ PCR product in a ligase-free PCR reaction. The desired overlap product was then amplified using nested primers (primers 2 and 6). The gene of interest was thereby absent in this final PCR amplicon, which was used for transformation of a highly competent population of *S. mutans* UA159. Briefly, *S. mutans* was grown overnight in peptide-free chemically defined medium (CDM)^53^, the cells were collected by centrifugation, washed twice and resuspended in PBS, and used to inoculate 500 μl peptide-free CDM broth at a dilution of 1:20. The cultures were grown to an OD_600_ of 0.1 when 1 μM of the competence pheromone ComX-inducing peptide (XIP) (GenScript, Piscataway, NJ) and 0.4 μg of the PCR amplicon were added to the culture. The cultures were incubated for an additional 3 hr before plating on BHI agar. After 48 hr incubation, colonies were screened by PCR using the primers listed in Table S3 to ensure that a double recombination event resulted in deletion of the target gene. The selected clone was confirmed by DNA sequencing of the PCR product. The *alsS* (*smu1492*) mutant strain was created by replacing the coding region of the gene with a non-polar kanamycin resistance cassette using a PCR ligation mutagenesis approach^54^. Briefly, PCR fragments flanking *alsS* were ligated to the kanamycin cassette and this ligation mix used to transform *S. mutans* UA159. Double mutants were obtained by amplifying the mutated *spxA1* region of the previously constructed Δ*spxA1* strain (spectinomycin-resistant, Spc^R^); this PCR product was then used to transform the newly generated single mutant strains.

### Growth and stress survival assays

To generate growth curves, strains were grown overnight under anaerobic conditions and diluted 1:20 in BHI or BHI containing 0.4 mM H_2_O_2_. The protective effects of α-ketoglutarate (KG), catalase, or selected amino acids on growth in the presence of H_2_O_2_ were tested by adding increasing concentrations of each reagent to the growth media. In all cases, cultures were incubated at 37 °C in a 5% CO_2_ atmosphere and the OD_600_ recorded at selected intervals. To test the sensitivity of *S. mutans* UA159 and its derivatives to H_2_O_2_ in disc diffusion assays, a uniform layer of exponentially-grown cells was spread using a sterile swab onto agar plates made of the chemically defined medium FMC^49^, or FMC lacking glycine, histidine, or glutamate. After spreading the bacterial cultures, Whatman filter paper discs (6 mm diameter) saturated with 20 μl of 0.5% H_2_O_2_ solution were placed on the agar and the diameter of the zone of growth inhibition measured after 24 hr incubation at 37 °C in 5% CO_2_. To test the ability of pre-loaded KG to protect *S. mutans*, BHI inocula were initiated in the presence or absence of 2 mM KG. Cells were grown to early log phase (OD_600_ = 0.3), then washed twice in phosphate-buffered saline to remove extracellular KG. These washed culture pellets were then resuspended in fresh BHI, either with or without 0.4 mM H_2_O_2_. Final growth yields were measured after 24 hr incubation. All stress survival assays were performed with quadruplicate culture replicates.

### Competition on solid media

Growth inhibition of *S. mutans* by production of H_2_O_2_ by mitis-group streptococci was performed as previously described^11^. Briefly, overnight cultures of *S. sanguinis* SK150 were normalized to OD_600_ of 0.5 and 8 μl aliquots spotted on BHI agar plates. After 16 hr incubation at 37 °C, overnight cultures of *S. mutans* UA159 and its derivatives were normalized to OD_600_ of 0.5 and 8 μl aliquots spotted next to the *S. sanguinis* spot. Plates were incubated for an additional 16 hr before visualizing the ability of the *S. mutans* strains to grow in proximity of the H_2_O_2_-generating *S. sanguinis*. To ensure that the growth inhibition was due to H_2_O_2_ production, a control condition included the addition of catalase directly on top of the *S. sanguinis* spot. Competition assays were performed with quadruplicate culture replicates.

### Metabolite profiling

Abundance of metabolic end products (lactate, ethanol, and formic acid) was measured using Megazyme enzymatic kits (Megazyme International, Wicklow, Ireland). Acetoin production was measured using a Voges-Proskauer test as described previously^55^. Cultures were grown in BHI medium to early log phase (OD_600_ = 0.35) and split into two equal volumes that were harvested by centrifugation after 60 additional minutes of growth: (A) unstressed control, (B) exposed to 0.5 mM H_2_O_2_. Culture supernatants were deproteinized by addition of 4 M perchloric acid, then neutralized with potassium hydroxide. The supernatants were then stored at −80 °C until detection of metabolic products according to the manufacturer’s protocol using a 96-well plate format. Results were normalized to colony-forming units recovered for each sample. All metabolite assays were performed with quadruplicate culture replicates.

### RNA analysis

Total RNA was isolated from homogenized *S. mutans* cell lysates by repeated hot acid-phenol:chloroform extractions as previously described^56^. The RNA was precipitated with ice-cold isopropanol and 3 M sodium acetate (pH 5) at 4 °C before RNA pellets were dissolved in nuclease-free H_2_O and treated with DNase I (Ambion, Carlsbad, CA) for 30 minutes at 37 °C. Then, 10 μg RNA aliquots were subjected to a second DNase I treatment using the DNA-free kit (Ambion). RNA concentrations were determined with the NanoDrop 1000 spectrophotometer (NanoDrop, Wilmington, DE) and RNA quality assessed with the Agilent Bioanalyzer (Agilent, Santa Clara, CA). RNA deep sequencing (RNA-Seq) was performed at the University of Rochester Genomics Research Center (UR-GRC) using the Illumina platform. The TruSeq RNA Sample Preparation Kit V2 (Illumina, San Diego, CA) was used for next generation sequencing library construction following the instructions from the manufacturer. The libraries were hybridized to the Illumina single end flow cell and amplified using cBot (Illumina) at a concentration of 8pM per lane. Raw reads were demultiplexed using configurebcl2fastq.pl version 1.8.4, and quality filtering and adapter removal performed using Trimmomatic version 0.32. Processed/cleaned reads were mapped to the *S. mutans* UA159 genome with STAR_2.4.2a. Initial differential expression analysis was performed using Cufflinks version 2.0.2, and DESeq. 2-1.10.1 was used for data normalization and differential expression analysis with an adjusted *p*-value threshold of 0.05^57^. Additional details on cDNA library construction, amplification and data analysis can be found at the UR-GRC website (https://www.urmc.rochester.edu/research/for-researchers/shared-resource-laboratories-facilities/laboratories/rochester-genomics-center.aspx). Gene expression trends as determined by RNA-Seq analysis were validated by qRT-PCR. Reverse transcription and qRT-PCR were carried out on triplicate samples of *S. mutans* UA159 RNA (unstressed or exposed to H_2_O_2_ as described for the RNA-Seq analysis) according to protocols described elsewhere^56^, using gene-specific primers listed in Table S6. Student’s *t* test was performed to verify significance of the qRT-PCR results. Gene expression data have been deposited in the NCBI Gene Expression Omnibus (GEO) database (www.ncbi.nlm.nih.gov/geo) under GEO Series Accession number GSE98526.

## Electronic supplementary material

Supplemental Data
