# Supplementary material for: Transcriptome responses of Streptococcus mutans to peroxide stress: identification of novel antioxidant pathways regulated by Spx
Source: Sci Rep. 2017 Nov 22;7:16018. doi: 10.1038/s41598-017-16367-5 (PMC5700188; doi:10.1038/s41598-017-16367-5)
Supplement: Supplementary file 1 — Supplemental Data [file 41598_2017_16367_MOESM1_ESM.pdf]

Transcriptome responses of *Streptococcus mutans* to peroxide stress: identification of novel antioxidant pathways regulated by Spx

Jessica K. Kajfasz, Tridib Ganguly, Emily L. Hardin, Jacqueline Abranches and José A. Lemos

Department of Oral Biology, University of Florida College of Dentistry, Gainesville FL, 32608, USA

Figure S1

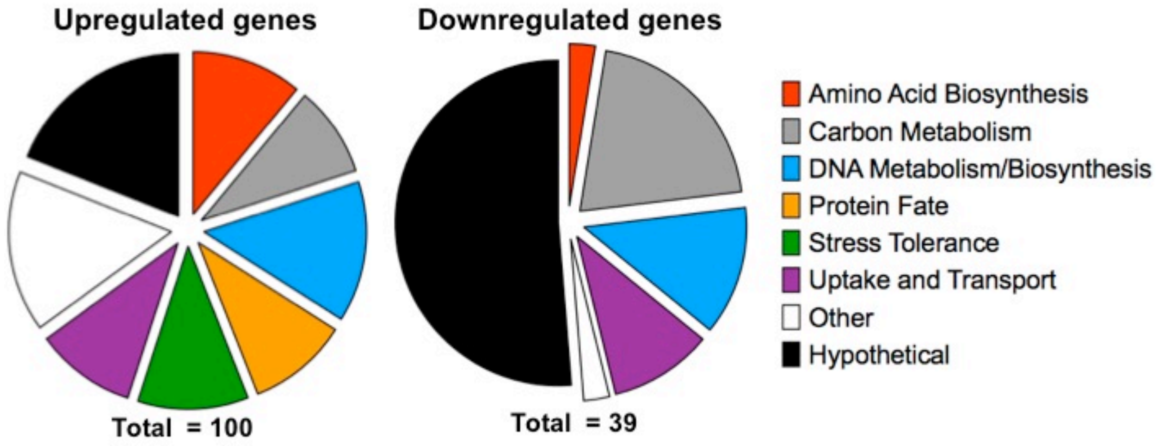

Fig. S1. Pie charts representing numbers of genes in various functional categories that were either up- or down-regulated in *S. mutans* UA159 after exposure to 0.4 mM H_2O_2 for 5 min as compared to an untreated control.

Figure S2

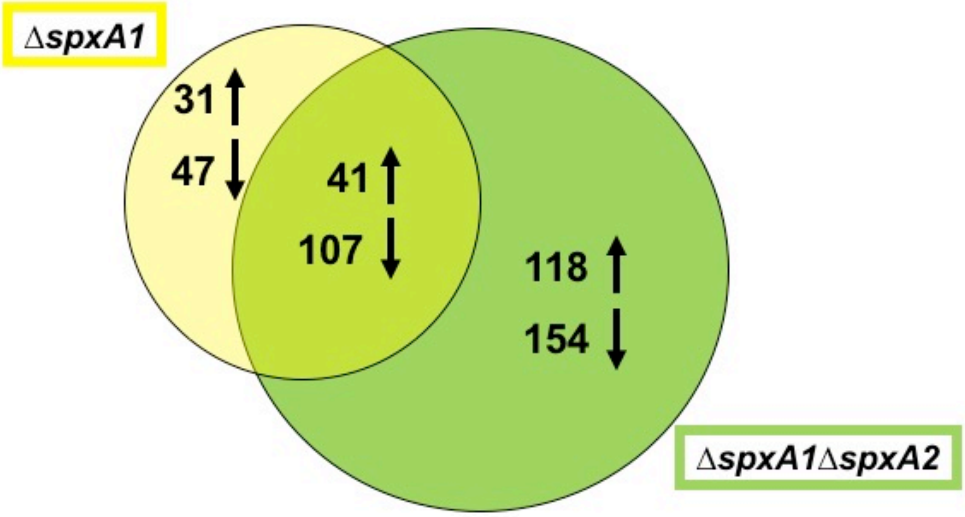

Fig. S2. Venn diagram depicting numbers of common and unique genes differently expressed in the Δ*spxA1* and Δ*spxA1*Δ*spxA2* strains subjected to peroxide stress when compared to the parent UA159 strain subjected to the same stress condition. (↑) number of genes upregulated, or (↓) downregulated as compared to UA159.

Table S1. List of genes differentially expressed following H_2O_2 exposure in *S. mutans* UA159 (*P* ≤ 0.05).

Gene	Description	Fold change H_2O_2/control	*p*-value
SMU_0018	hypothetical protein	-11.211	3.09E-06
SMU_0021	mreD, putative cell shape-determining protein	-2.470	3.77E-03
SMU_0061	transcriptional regulator, Cro/CI family	2.386	1.21 E-03
SMU_0114	putative PTS system, fructose-specific IIA component	-4.164	5.50 E-04
SMU_0124	putative transcriptional regulator (MarR family)	2.935	2.71 E-03
SMU_0125	conserved hypothetical protein	2.424	4.30E-03
SMU_0127	adhA, acetoin dehydrogenase E1 component alpha subunit	4.110	1.22E-08
SMU_0128	adhB, acetoin dehydrogenase E1 component beta subunit	4.597	1.76E-06
SMU_0129	adhC, dihydrolipoamide S-acetyltransferase	6.443	2.73E-13
SMU_0130	adhD, dihydrolipoamide dehydrogenase	7.264	1.09E-14
SMU_0131	lplA, lipoate-protein ligase	6.913	6.53E-14
SMU_0132	hipO, amino acid amidohydrolase (hippurate amidohydrolase)	4.205	3.25E-05
SMU_0143c	def, polypeptide deformylase (PDF)	3.269	4.12E-05
SMU_0144c	possible transcriptional regulator	2.824	1.94 E-03
SMU_0187c	dusA, tRNA-dihydrouridine synthase	2.785	2.40E-03
SMU_0188c	hslO, putative 33 kD chaperonin (heat shock protein)	3.096	1.63E-03
SMU_0189	hypothetical protein	-10.595	1.50E-09
SMU_0196c	immunogenic secreted protein (transfer protein)	-3.277	2.57E-04
SMU_0197c	hypothetical protein	-3.729	1.59E-04
SMU_0199c	hypothetical protein	-41.579	0
SMU_0202c	conserved hypothetical protein/Streptococcus-specific protein	-17.081	2.62E-04
SMU_0206c	hypothetical protein	-28.301	1.28E-04
SMU_0208c	putative transposon protein	-23.292	0
SMU_0209c	hypothetical protein	-13.294	9.25E-04
SMU_0211c	hypothetical protein	-63.887	1.01E-06
SMU_0212c	hypothetical protein	-696.534	3.44E-04
SMU_0247	sufC, ABC transporter, ATP-binding protein	4.714	6.63E-10
SMU_0248	sufD, protein fate	4.753	5.37E-10
SMU_0249	sufS, class-V aminotransferase	5.043	2.41E-08
SMU_0250	sufU, putative nitrogen fixation-like protein	4.480	3.17E-03
SMU_0251	sufB	3.574	5.33E-07
SMU_0267	gcl, glutamate--cysteine ligase	2.083	3.59E-03
SMU_0277	hypothetical protein	-2.575	1.27E-03
SMU_0328	putative carbonic anhydrase	2.942	1.10E-05
SMU_0329	conserved hypothetical protein	2.941	1.27E-05
SMU_0363	glnR, transcriptional regulator; glutamine synthetase repressor	-4.377	1.74E-07
SMU_0364	glnA, glutamine synthetase type 1	-2.804	2.33E-04
SMU_0378	hypothetical protein	-6.601	3.47E-03
SMU_0438c	NADPH-dependent glutamate synthase (small subunit)	-3.434	8.17E-06

SMU_0458	rheB, putative ATP-dependent RNA helicase	2.184	1.65E-03
SMU_0463	trxB, thioredoxin reductase	3.821	5.55E-08
SMU_0478	gmk, putative guanylate kinase	2.195	2.72E-03
SMU_0496	cysK, cysteine synthetase A	3.780	2.56E-07
SMU_0562	clpE, ATP-dependent protease	3.321	1.04E-04
SMU_0570	feoB, ferrous ion transport protein B	-2.973	1.30E-04
SMU_0626	comEC, putative competence protein	-8.701	5.73E-09
SMU_0629	sodA, superoxide dismutase	5.119	1.07E-08
SMU_0667	nrdF, ribonucleotide reductase, small subunit	2.929	3.78E-05
SMU_0668c	nrdA, ribonucleotide reductase, large subunit	2.905	3.08E-05
SMU_0670	citB, aconitate hydratase aconitase	-3.062	7.09E-05
SMU_0676	gapN, NADP-dependent glyceraldehyde-3-phosphate dehydrogenase	2.766	4.45E-04
SMU_0725c	conserved hypothetical protein	3.115	1.84E-03
SMU_0764	ahpC, alkyl hydroperoxide reductase, subunit C	11.528	4.44E-16
SMU_0765	ahpF, alkyl hydroperoxide reductase, subunit F	10.597	2.44E-14
SMU_0838	gor, glutathione reductase	5.968	1.21E-11
SMU_0841	folC, putative folyl-polyglutamate synthetase	2.343	7.19E-04
SMU_0842	conserved hypothetical protein	2.058	3.22E-03
SMU_0875c	Tpn, putative transposase, IS150-like	-5.025	1.74E-04
SMU_0924	tpx, thiol peroxidase	7.366	6.33E-12
SMU_0929c	conserved hypothetical protein	3.184	4.13E-05
SMU_0956	clpL, ATP-dependent Clp protease, ATP-binding subunit	2.525	1.23E-03
SMU_0980	bglP, beta-glucoside-specific EII permease	-5.551	6.52E-06
SMU_0981	bglB, putative BglB fragment	-9.56-	3.79E-03
SMU_0991	putative ribonucleotide reductase	2.992	7.92E-04
SMU_1001	dprA, DNA processing protein, Smf family	-25.404	8.99E-04
SMU_1048	conserved hypothetical protein	4.530	2.63E-03
SMU_1062	bet, putative ABC transporter, proline/glycine betaine permease protein	2.748	5.34E-05
SMU_1063	atmD, putative ABC transporter, ATP-binding protein, proline/glycine betaine	2.694	5.56E-05
SMU_1071c	conserved hypothetical protein	3.198	2.92E-03
SMU_1073	fhs, formate--tetrahydrofolate ligase	2.493	2.75E-03
SMU_1116c	hypothetical protein	3.604	5.34E-06
SMU_1117	nox, H2O-forming NADH Oxidase	4.6568	4.28E-06
SMU_1124	pdp, putative pyrimidine-nucleoside phosphorylase	-2.210	4.23E-03
SMU_1147c	hypothetical protein	-12.283	3.04E-03
SMU_1264	hisF, putative phosphoribosyl-ATP pyrophosphatase / phosphoribosyl-AMP cyclohydrolase	3.706	3.49E-03
SMU_1265	hisA, putative imidazoleglycerol-phosphate synthase, cyclase subunit	5.072	6.40E-04
SMU_1266	hisH, putative phosphoribosyl aminoimidazole carboxamide ribonucleotide isomerase	6.371	4.35E-04
SMU_1267c	hypothetical protein	3.043	1.89E-03
SMU_1269	serB, putative imidazoleglycerol-phosphate dehydratase	4.885	4.04E-03
SMU_1270	hisD, putative phosphoserine phosphatase	5.825	1.19E-04
SMU_1272	hisZ, putative histidyl-tRNA synthetase	7.003	9.02E-05
SMU_1273	hisC, putative ATP phosphoribosyltransferase	5.038	2.72E-04
SMU_1296	yghU, glutathione S-transferase	4.803	2.23E-03
SMU_1297	conserved hypothetical protein, DHH family	5.072	3.10E-06
SMU_1370c	orfY, transposase, IS861, IS3 family	-5.004	1.81E-04
SMU_1394	lepA, putative GTP-binding protein	2.104	3.56E-03

SMU_1396	gbpC, glucan-binding protein C	3.138	1.95E-04
SMU_1411	conserved hypothetical protein	-4.908	3.37E-05
SMU_1451	aldB, alpha-acetolactate decarboxylase	3.647	8.97E-06
SMU_1452	alsS, alpha-acetolactate synthase	2.894	2.75E-05
SMU_1491	lacE, PTS system, lactose-specific enzyme IIA	-4.470	6.88E-05
SMU_1495	lacB, galactose-6-phosphate isomerase	-8.559	6.78E-04
SMU_1504c	hypothetical protein	-4.096	7.66E-04
SMU_1505c	hypothetical protein	-10.732	3.82E-03
SMU_1519	glnQ, putative amino acid ABC transporter, ATP-binding protein	-2.893	1.54E-03
SMU_1525	murA,putative UDP-N-acetylglucosamine 1-carboxyvinyltransferase	2.250	1.38E-03
SMU_1561	trkB, putative potassium uptake system protein	2.885	8.39E-04
SMU_1562	trkA, putative potassium uptake protein	2.987	5.82E-05
SMU_1563	pacL, putative cation-transporting P-type ATPase	2.604	9.76E-05
SMU_1566	malR, maltose operon transcriptional repressor	2.849	4.90E-05
SMU_1574c	conserved hypothetical protein	2.260	3.85E-03
SMU_1593c	conserved hypothetical protein	2.295	2.79E-03
SMU_1596	celB, PTS system, cellobiose-specific IIC component	-5.335	4.38E-04
SMU_1645	tehB, tellurite resistance protein	2.681	9.54E-05
SMU_1649	exoA, exodeoxyribonuclease III/ Smx nuclease	3.085	3.52E-05
SMU_1664c	cobQ, putative cobyric acid synthase	2.133	2.45E-03
SMU_1668	livH, putative branched chain amino acid ABC transporter	2.350	6.19E-04
SMU_1669	livK, putative ABC transporter, branched chain amino acid-binding	2.801	5.52E-04
SMU_1692	pflA, pyruvate-formate lyase activating enzyme	3.553	1.08E-06
SMU_1722c	putative integral membrane protein	2.259	2.71E-03
SMU_1760c	conserved hypothetical protein	4.814	2.86E-06
SMU_1761c	conserved hypothetical protein	6.529	3.21E-06
SMU_1763c	conserved hypothetical protein	5.495	2.43E-04
SMU_1764c	conserved hypothetical protein	6.066	6.07E-10
SMU_1787c	preprotein translocase, YajC subunit	2.088	2.99E-03
SMU_1788c	bta, bacterocin transport accessory protein	4.539	9.04E-07
SMU_1814	scnK, histidine kinase	4.092	2.30E-08
SMU_1815	scnR, response regulator	4.487	9.00E-06
SMU_1818c	hypothetical protein	-16.312	3.32E-04
SMU_1849	comEB, putative deoxycytidylate deaminase	3.781	2.77E-05
SMU_1851	uvrA, putative excinuclease ABC (subunit A)	4.035	4.35E-06
SMU_1865	mutY, A/G-specific adenine glycosylase	3.120	4.31E-04
SMU_1867c	adhB, putative alcohol dehydrogenase	3.653	1.37E-07
SMU_1869	trxA, thioredoxin	4.814	4.73E-10
SMU_1900	comB, conserved hypothetical protein	-8.159	6.63E-11
SMU_1916	comD, histidine kinase of the competence regulon	4.394	5.89E-06
SMU_1917	comE, response regulator	4.627	7.16E-05
SMU_1942c	atmA, putative amino acid binding protein	3.176	4.86E-06
SMU_1954	groEL, chaperonin	2.599	1.07E-03
SMU_1955	groES, co-chaperonin	2.977	4.00E-05
SMU_1960c	levE, PTS system, sugar-specific enzyme IIA component	-2.327	1.67E-03
SMU_1961c	levD, PTS system, trehalose-specific IIABC component	-2.290	1.41E-03
SMU_1988c	probable DNA binding protein	4.903	3.77E-04
SMU_2027	transcriptional regulator/repressor	4.353	1.61E-03
SMU_2036	PepO, endopeptidase O	2.580	8.06E-04

SMU_2044	relA, conserved hypothetical protein	3.396	9.70E-07
SMU_2102	hisS, histidine-tRNA synthetase	2.740	4.68E-05
SMU_2142	rpiA, putative sugar-phosphate isomerase	2.477	1.62E-03
SMU_2143c	trmU, putative tRNA	2.487	2.48E-04

Table S2. List of genes differentially expressed following H_2O_2 exposure in *S. mutans Δspx* strains as compared to the peroxide regulon of wild type strain UA159 ($P \leq 0.05$).

Gene	Description	Fold change Δ*spxA1*/UA159	*p*-value	Fold change Δ*spxA2*/UA159	*p*-value	Fold change Δ*spxA1*Δ*spxA2*/ UA159	*p*-value
SMU_0009	Hypothetical			-2.654	3.70E-03		
SMU_0013	Cell division/envelope			2.665	4.17E-03	2.721	5.31E-04
SMU_0016	Transport and binding	-2.134	2.18E-03				
SMU_0024	aspC, Biosynthesis: Macromolecules and co-factors	2.112	2.69E-03				
SMU_0039	Hypothetical					2.934	7.79E-04
SMU_0043	DNA metabolism					-2.225	7.50E-04
SMU_0044	Hypothetical	-2.071	4.03E-03				
SMU_0045	Hypothetical	-2.437	9.61E-05			-2.389	9.61E-05
SMU_0060	alkD, DNA metabolism					3.211	4.08E-06
SMU_0061	Transcription	-5.306	9.02E-14			-4.340	9.02E-14
SMU_0071	Stress tolerance/detoxification					-2.890	5.61E-06
SMU_0080	hrcA, Transcription					2.712	7.99E-06
SMU_0081	grpE, Protein fate					2.682	9.65E-05
SMU_0082	dnaK, Protein fate					2.075	5.75E-04
SMU_0084	truA, Biosynthesis: Macromolecules and co-factors	2.762	2.47E-03				
SMU_0087	Hypothetical	2.571	1.89E-04			2.339	1.89E-04
SMU_0089c	Transport and binding	-2.743	2.18E-04			-3.946	2.18E-04
SMU_0092c	tpn, Hypothetical					2.505	4.66E-04
SMU_0105	scrR, Transcription					2.111	2.69E-03
SMU_0110	Transcription	2.224	1.47E-03				
SMU_0119	adh, Energy metabolism					-2.234	1.65E-03
SMU_0120	rpmB, Biosynthesis: Macromolecules and co-factors					2.285	3.26E-03
SMU_0124	ykoM, Transcription	-2.16	8.45E-04			-3.326	8.45E-04
SMU_0125	Hypothetical	-2.336	1.77E-04			-3.965	1.77E-04

SMU_0127	adhA. Engery metabolism	-11.171	0			-22.109	0
SMU_0128	adhB, Engery metabolism	-12.093	0			-21.177	0
SMU_0129	adhC, Engery metabolism	-11.518	0			-17.827	0
SMU_0130	adhD, Engery metabolism	-10.566	0			-15.575	0
SMU_0131	lplA, Other	-10.559	0			-16.015	0
SMU_0132	hipO, Central metabolism	-6.478	4.44E-16			-8.168	4.44E-16
SMU_0133c	Transport and binding	-2.878	6.30E-05			-4.182	6.30E-05
SMU_0135	mleR, Transcription	3.336	8.38E-07			2.292	8.38E-07
SMU_0136c	Hypothetical	3.086	01.31E-04				
SMU_0137	mleS, Energy metabolism	-5.095	3.91E-10	-2.896	3.91E-10	-7.283	3.91E-10
SMU_0138	mleP, Transport and binding	-6.331	2.72E-10			-10.406	2.72E-10
SMU_0139	oxdC, Central metabolism	-6.974	1.55E-11			-3.599	1.55E-11
SMU_0140	gor, Redox homeostasis	-5.289	6.68E-10			-3.367	6.68E-10
SMU_0141	Hypothetical	-6.445	7.63E-10			-3.726	7.63E-10
SMU_0143c	def, Protein fate	-3.627	6.13E-09			-4.463	6.13E-09
SMU_0144c	Transcription	-6.157	7.33E-15			-7.311	7.33E-15
SMU_0145	Hypothetical					2.066	1.94E-03
SMU_0148	adhE, Energy metabolism	-1.934	2.80E-03				
SMU_0187c	dusA, Biosynthesis: Macromolecules and co-factors					-4.978	1.96E-08
SMU_0188c	hslO, Protein fate					-5.072	3.46E-08
SMU_0189	Hypothetical			3.401	7.66E-04		
SMU_0199c	Hypothetical	3.593	4.33E-04				
SMU_0202c	Hypothetical	4.73	1.64E-03				
SMU_0207c	Hypothetical	3.64	2.01E-04				
SMU_0208c	Hypothetical	3.867	5.61E-06	3.251	5.61E-06		
SMU_0209c	Hypothetical	5.484	2.79E-04				
SMU_0210c	Hypothetical	7.495	3.12E-04				
SMU_0211c	Hypothetical	8.691	1.82E-03				
SMU_0224c	Hypothetical					2.596	8.22E-05
SMU_0226c	tpn, DNA metabolism					1.926	3.60E-03
SMU_0227c	Hypothetical					2.246	4.20E-04
SMU_0229	Hypothetical					-2.011	2.97E-03
SMU_0231	ilvB, Biosynthesis: Macromolecules and co-factors					2.645	1.36E-04
SMU_0234	ilvA, Biosynthesis:					-2.483	7.13E-06

	Macromolecules and co-factors						
SMU_0247	sufC, Transport and binding	-4.158	4.26E-12			-7.036	4.26E-12
SMU_0248	sufD, Protein fate	-4.315	1.12E-12			-7.552	1.12E-12
SMU_0249	sufS, Other	-4.367	5.32E-10			-7.494	5.32E-10
SMU_0250	sufU, Other	-3.98	1.14E-03			-6.959	1.14E-03
SMU_0251	sufB	-4.163	9.35E-12			-6.905	9.35E-12
SMU_0252	Hypothetical	-2.055	4.02E-03				
SMU_0253	dacA, Cell division/envelope					2.0626	3.34E-03
SMU_0255	oppA, Transport and binding					2.332	8.24E-05
SMU_0260	Hypothetical	-4.214	5.53E-09			-6.238	5.53E-09
SMU_0262	aguB , Other					-3.574	6.64E-05
SMU_0263	aguD, Transport and binding					-4.840	1.68E-08
SMU_0264	aguA, Central metabolism					-5.370	3.76E-07
SMU_0265	aguC, Central metabolism					-4.009	8.87E-05
SMU_0267c	gcl, Central metabolism	1.900	2.77E-03			-3.809	2.77E-03
SMU_0270	sgaT, Signal transduction					2.356	5.69E-04
SMU_0271	ptxB, Signal transduction	-2.800	3.59E-05			-2.363	3.59E-05
SMU_0272	ptxA, Transport and binding	-3.896	7.92E-08			-3.367	7.92E-08
SMU_0273	rmpD, Energy metabolism	-3.330	4.83E-07			-3.430	4.83E-07
SMU_0274	rmpE, Energy metabolism	-2.848	2.19E-05			-2.378	2.19E-05
SMU_0275	rmpF, Energy metabolism	-2.666	4.59E-05			-2.181	4.59E-05
SMU_0277	Hypothetical	3.252	1.37E-06	2.692	1.37E-06	4.525	1.37E-06
SMU_0278	Hypothetical	3.069	6.44E-06			4.152	6.44E-06
SMU_0279	Hypothetical					4.464	2.82E-05
SMU_0281	Hypothetical	2.738	1.30E-04			3.951	1.30E-04
SMU_0283	Hypothetical					4.492	3.72E-08
SMU_0284	Hypothetical					3.199	6.68E-05
SMU_0285	Hypothetical	3.201	9.19E-06			4.859	9.19E-06
SMU_0287	comB, Competence					2.131	4.42E-04
SMU_0294	Hypothetical					4.836	3.25E-06
SMU_0295	Hypothetical					4.155	3.86E-05
SMU_0296	Hypothetical					3.421	5.01E-07
SMU_0297	polA, DNA metabolism					-4.501	2.48E-13
SMU_0298	Hypothetical					-4.340	2.09E-11
SMU_0322c	galU, Biosynthesis:					-2.920	1.46E-06

	Macromolecules and co-factors						
SMU_0323	gpdA, Central Metabolism					-3.260	2.65E-08
SMU_0328	Central metabolism	-2.037	4.54E-04			-6.773	4.54E-04
SMU_0329	Hypothetical	-1.886	1.81E-03			-3.177	1.81E-03
SMU_0333	Hypothetical						
SMU_0334	argG, Biosynthesis: Macromolecules and co-factors			2.456	2.10E-03	3.192	2.10E-03
SMU_0335	argH, Biosynthesis: Macromolecules and co-factors					2.128	1.54E-03
SMU_0348	Hypothetical	-3.078	2.57E-06			-4.646	2.57E-06
SMU_0349	ksgA, Other	-3.732	6.15E-09			-5.258	6.15E-09
SMU_0350	Hypothetical	-3.428	1.36E-05			-3.813	1.36E-05
SMU_0367	Hypothetical					1.984	1.71E-03
SMU_0381c	Hypothetical					-1.983	9.10E-04
SMU_0383c	Hypothetical					2.725	1.52E-03
SMU_0384	Hypothetical					-3.288	3.92E-06
SMU_0385	Protein fate					-3.163	4.93E-04
SMU_0386	rimI, Biosynthesis: Macromolecules and co-factors					-2.916	1.13E-03
SMU_0387	gcp, Protein fate					-2.821	2.26E-05
SMU_0393	Hypothetical	2.044	4.13E-03			2.657	4.13E-03
SMU_0394c	Hypothetical					2.694	9.53E-05
SMU_0396	glpF, Transport and binding					2.106	5.77E-04
SMU_0400	Pathogenesis					-2.335	4.14E-03
SMU_0402	pfl, Central Metabolism	-2.029	1.15E-03				
SMU_0403	dinP, DNA metabolism					-3.702	4.40E-09
SMU_0404c	Hypothetical					-2.516	6.47E-04
SMU_0405c	Transcription					-3.306	1.25E-04
SMU_0408	Transport and binding					2.450	3.70E-05
SMU_0423	nlmD, Pathogenesis					2.378	4.85E-04
SMU_0431	cylA, Transport and binding			7.269	1.02E-08	26.280	1.02E-08
SMU_0432	cylB, Transport and binding			6.237	4.72E-07	17.574	4.72E-07
SMU_0433	Hypothetical					2.953	7.53E-06
SMU_0434	Hypothetical			2.336	4.09E-03	2.403	4.09E-03
SMU_0438c	Hypothetical			2.771	8.62E-04	2.715	8.62E-04
SMU_0439	Transcription					2.410	4.35E-03

SMU_0458	rheB, Biosynthesis: Macromolecules and co-factors	-2.684	2.13E-06	-3.203	2.13E-06
SMU_0463	trxB, Stress tolerance/detoxification	-4.713	7.53E-14	-6.411	7.53E-14
SMU_0478	gmk, Biosynthesis: Macromolecules and co-factors			-1.852	3.93E-03
SMU_0479	rpoZ, Transcription			-1.910	1.85E-03
SMU_0480	priA, DNA metabolism	-2.103	2.75E-04	-2.472	2.75E-04
SMU_0501	Hypothetical			3.687	4.44E-08
SMU_0502	Hypothetical	1.88	4.23E-03	3.3	4.23E-03
SMU_0503c	Hypothetical	2.556	9.06E-04	2.901	9.06E-04
SMU_0510c	Hypothetical			2.366	3.35E-04
SMU_0514	Hypothetical			2.149	2.05E-03
SMU_0515	mycA, Pathogenesis			2.768	5.15E-05
SMU_0522	Redox homeostasis			-2.735	2.29E-03
SMU_0524	Transport and binding	-4.535	7.30E-06	-4.508	7.30E-06
SMU_0525	Transport and binding	-4.447	1.48E-06	-4.446	1.48E-06
SMU_0526c	Transcription			-3.063	4.16E-06
SMU_0527	Hypothetical			-2.419	2.55E-04
SMU_0540	dpr, Stress tolerance/detoxification	-4.450	1.98E-05	-14.183	1.98E-05
SMU_0542	glk, Energy metabolism	-1.935	1.80E-03		
SMU_0546	bipA, Biosynthesis: Macromolecules and co-factors			-2.181	9.18E-04
SMU_0547	Hypothetical			-2.505	3.08E-05
SMU_0562	clpE, Protein fate	-2.569	1.32E-04	-5.278	1.32E-04
SMU_0569	feoA, Transport and binding	6.135	2.03E-03	5.824	2.03E-03
SMU_0570	feoB, Transport and binding	4.930	6.70E-11	5.187	6.70E-11
SMU_0571	Hypothetical	4.734	9.53E-08	5.536	9.53E-08
SMU_0575c	lrgA, Signal transduction	-3.174	4.06E-03		
SMU_0580	xseA, DNA metabolism			-2.794	1.55E-03
SMU_0583	hlyX, Other			-3.314	1.43E-03
SMU_0585	recN, DNA metabolism			-3.232	1.99E-08
SMU_0589	hlpA, DNA metabolism			-2.008	6.36E-04
SMU_0593	furR, Transcription	-2.629	1.16E-04	-3.939	1.16E-04
SMU_0602	Transport and binding			2.4202	2.56E-04
SMU_0609	Bsp, Cell envelope			3.138	2.67E-06

SMU_0610	spaP, Pathogenesis			-10.966	4.32E-03
SMU_0611	deaD, Biosynthesis: Macromolecules and co-factors			-1.995	7.20E-04
SMU_0616	Hypothetical			2.767	2.22E-06
SMU_0618	Hypothetical			2.915	3.02E-06
SMU_0629	sodA, Stress tolerance/detoxification	-15.253	0	-50.797	0
SMU_0630	Hypothetical	-2.082	1.60E-03		
SMU_0634	queA, Biosynthesis: Macromolecules and co-factors	-2.115	5.58E-04	-2.067	5.58E-04
SMU_0635	Hypothetical			-3.417	2.97E-09
SMU_0636	nagB, Central metabolism			-4.365	7.07E-13
SMU_0637c	Hypothetical	-2.520	6.78E-05	-6.338	6.78E-05
SMU_0638	rsuA, Biosynthesis: Macromolecules and co-factors			-2.498	2.38E-04
SMU_0639	rimJ, Biosynthesis: Macromolecules and co-factors			-2.209	3.28E-03
SMU_0640c	ydeF, Transcription			-2.287	5.77E-04
SMU_0645	pepB, Protein fate			-2.503	4.69E-05
SMU_0646	gph, Other			-2.415	1.99E-03
SMU_0647	Other			-2.537	1.36E-04
SMU_0654	Transport and binding	-5.013	6.94E-05		
SMU_0656	Transport and binding	-6.848	4.28E-04		
SMU_0657	mutG, Transport and binding	-5.200	2.14E-05	-4.989	2.14E-05
SMU_0663	argC, Biosynthesis: Macromolecules and co-factors	-4.922	1.65E-07	-3.948	1.65E-07
SMU_0664	argJ, Biosynthesis: Macromolecules and co-factors	-4.126	1.98E-07	-3.767	1.98E-07
SMU_0665	argB, Biosynthesis: Macromolecules and co-factors	-3.553	7.95E-06	-2.603	7.95E-06
SMU_0666	argD, Biosynthesis: Macromolecules and co-factors	-5.388	1.47E-09	-4.685	1.47E-09
SMU_0667	nrdF, Biosynthesis: Macromolecules and co-factors	-4.186	3.81E-11	-6.126	3.81E-11
SMU_0668c	nrdA, Central metabolism	-5.288	1.55E-15	-8.025	1.55E-15
SMU_0669c	nrdH, Redox homeostasis	-3.720	1.44E-07	-11.889	1.44E-07
SMU_0670	citB, Energy metabolism			-3.791	1.41E-07

SMU_0671	citZ, Energy metabolism	-2.203	1.79E-03			-4.978	1.79E-03
SMU_0672	citC, Energy metabolism	-2.482	3.05E-04			-5.180	3.05E-04
SMU_0673	Hypothetical	-1.981	3.90E-03			-3.278	3.90E-03
SMU_0691	pepT, Protein fate					2.598	2.58E-05
SMU_0704c	Cell division/envelope					-2.770	6.61E-05
SMU_0707c	Cell division/envelope	-2.201	1.10E-04			-2.174	1.10E-04
SMU_0709	Hypothetical					3.904	1.95E-06
SMU_0711	Hypothetical					3.714	1.97E-03
SMU_0713	ftsW, Cell division/envelope					-2.003	6.27E-04
SMU_0720	Hypothetical					2.338	3.87E-04
SMU_0721	Hypothetical					2.044	4.12E-03
SMU_0724	glpQ, Central metabolism					-2.936	8.62E-05
SMU_0725c	Hypothetical	-2.098	1.54E-03			-8.412	1.54E-03
SMU_0728	Redox homeostasis					-2.734	6.62E-04
SMU_0730	Hypothetical	2.2502	4.07E-03			2.775	4.07E-03
SMU_0745	Stress tolerance/detoxification					2.391	2.32E-04
SMU_0746c	Hypothetical					-4.012	1.88E-07
SMU_0747c	Transport and binding					-3.772	3.68E-05
SMU_0753	Hypothetical	2.0717	1.03E-03	5.172	1.03E-03	7.612	1.03E-03
SMU_0764	ahpC, Stress tolerance/detoxification	-20.554	0			-74.797	0
SMU_0765	ahpF, Redox homeostasis	-20.912	0			-70.199	0
SMU_0772	gbpD, Pathogenesis					2.690	2.95E-05
SMU_0775c	Hypothetical					-1.8005	4.38E-03
SMU_0802	Hypothetical					-2.775	3.25E-04
SMU_0815	Transport and binding					2.0213	2.56E-03
SMU_0817	glnH, Transport and binding					2.030	2.52E-03
SMU_0819	mscL, Transport and binding	-2.908	3.66E-06				
SMU_0836	Hypothetical					-2.279	1.69E-03
SMU_0838	gor, Stress tolerance/detoxification	-5.435	1.11E-15			-11.175	1.11E-15
SMU_0841	Biosynthesis: Macromolecules and co-factors	-2.060	5.31E-04			-3.502	5.31E-04
SMU_0842	thiI, Biosynthesis: Macromolecules and co-factors	-2.159	1.59E-04			-3.648	1.59E-04
SMU_0876	msmR, Transcription					3.334	2.77E-06
SMU_0877	aga, Energy metabolism					3.408	4.36E-07

SMU_0878	msmE, Transport and binding			3.202	4.59E-06
SMU_0879	msmF, Transport and binding			2.853	3.05E-05
SMU_0880	msmG, Transport and binding			2.658	1.28E-04
SMU_0881	gtfA, Energy metabolism			2.436	2.18E-04
SMU_0882	msmK, Transport and binding			2.317	5.93E-04
SMU_0883	dexB, Energy metabolism			2.404	2.05E-04
SMU_0886	galK, Energy metabolism			2.864	5.91E-05
SMU_0887	galT, Energy metabolism			2.066	2.57E-03
SMU_0913	gdhA, Central metabolism	1.858	3.59-03	2.349	3.59E-03
SMU_0914c	Hypothetical			2.475	4.92E-04
SMU_0915c	Central metabolism			2.392	9.56E-04
SMU_0921	Transcription			-2.467	5.53E-05
SMU_0922	Transport and binding			-2.313	3.18E-03
SMU_0924	tpx, Stress tolerance/detoxification	-40.003	0	-120.397	0
SMU_0925	Hypothetical	-3.3607	2.61E-07	-2.384	2.61E-07
SMU_0929c	Hypothetical	-13.921	0	-19.523	0
SMU_0932	Hypothetical	3.494	4.64E-07		
SMU_0933	atmA, Transport and binding	3.695	8.92E-08		
SMU_0934	Transport and binding	3.607	2.73E-07		
SMU_0935	Transport and binding	4.498	1.55E-09		
SMU_0936	Transport and binding	3.624	6.56E-07		
SMU_0942	mvaA, Other			-8.918	4.62E-14
SMU_0943c	mvaS, Central metabolism			-8.177	7.56E-13
SMU_0944	thyA, Biosynthesis: Macromolecules and co-factors			-4.300	6.26E-10
SMU_0946	pnuC, Transport and binding			-2.873	2.57E-05
SMU_0947	dfr, Biosynthesis: Macromolecules and co-factors			-11.399	0
SMU_0948	Hypothetical			-6.605	4.09E-06
SMU_0949	clpX, Protein fate			-10.929	0
SMU_0950	era, Other			-10.966	0
SMU_0951	rocE, Transport and binding			-2.703	2.30E-05
SMU_0952	mmuM, Energy metabolism			-3.170	2.95E-06
SMU_0956	clpL, Protein fate	-1.900	3.63E-03		
SMU_0961	Hypothetical	6.250	2.62E-14	2.734	2.62E-14
SMU_0962	mmgC, Central metabolism	4.712	2.24E-11	2.029	2.24E-11

SMU_0984	Hypothetical					5.574	6.22E-11
SMU_0991	Redox homeostasis	-3.394	1.18E-07			-4.642	1.18E-07
SMU_0992	Hypothetical					2.993	3.41E-06
SMU_0993	ylqL, Other					2.678	4.24E-04
SMU_0995	yclN, Transport and binding	14.552	7.03E-10			15.896	7.03E-10
SMU_0996	yclN, Transport and binding	16.433	8.69E-08			21.493	8.69E-08
SMU_0997	fecE, Transport and binding	16.932	6.60E-08			17.043	6.60E-08
SMU_0998	fatB, Transport and binding	18.755	0			19.535	0
SMU_0999	Hypothetical					4.316	6.11E-08
SMU_1002	topA, DNA metabolism					-1.987	1.12E-03
SMU_1003	gidA, Cell division/envelope	-2.056	1.36E-03			-2.338	1.36E-03
SMU_1008	llrG, Signal transduction					-3.269	1.69E-04
SMU_1013c	yxiQ, Transport and binding					-2.136	3.03E-03
SMU_1027	Transcription	2.606	4.43E-04			2.256	4.43E-04
SMU_1028	Central Metabolism	2.324	7.03E-04				
SMU_1042	Hypothetical					2.069	4.21E-03
SMU_1046c	relQ, Stress tolerance/detoxification					-2.486	3.66E-04
SMU_1048	Hypothetical	-5.622	3.77E-12			-3.661	3.77E-12
SMU_1062	busAB, Transport and binding					-2.278	5.19E-05
SMU_1063	atmD, Transport and binding					-2.993	7.33E-08
SMU_1064c	busR, Transcription					2.296	9.52E-04
SMU_1065c	rgrB, Transcription					2.420	4.99E-04
SMU_1067c	Transport and binding					6.641	3.65E-08
SMU_1068c	Transport and binding			3.498	1.06E-03	7.606	1.06E-03
SMU_1069c	Hypothetical					6.668	2.92E-10
SMU_1070c	Hypothetical					8.742	7.98E-09
SMU_1071c	Hypothetical	-3.964	7.13E-09			-4.885	7.13E-09
SMU_1073	fhs, Biosynthesis: Macromolecules and co-factors	-2.568	3.44E-05			-2.468	3.44E-05
SMU_1091	wapE, Cell division/envelope					1.925	2.26E-03
SMU_1109c	Hypothetical					2.385	2.10E-047
SMU_1116c	Hypothetical	-6.751	0			-3.956	0
SMU_1117	nox, Stress tolerance/detoxification	-19.859	0			-17.065	0
SMU_1124	pdp, Biosynthesis:					2.388	2.11E-04

SMU_1125c	Macromolecules and co-factors Hypothetical				2.365	4.00E-04
SMU_1127	rpsT, Biosynthesis: Macromolecules and co-factors	2.212	3.81E-03			
SMU_1131c	Hypothetical				4.359	2.88E-06
SMU_1139c	yebU, Biosynthesis: Macromolecules and co-factors	-2.101	3.46E-03		-2.111	3.46E-03
SMU_1140c	Hypothetical				-2.522	8.95E-05
SMU_1142c	spxA1, Transcription	-1568.7503	1.96E-05			
SMU_1143c	mreA, Biosynthesis: Macromolecules and co-factors				-2.437	8.28E-05
SMU_1144	truB, Biosynthesis: Macromolecules and co-factors				-2.263	2.23E-04
SMU_1153c	Hypothetical	-2.123	3.08E-03			
SMU_1164c	Transport and binding				2.613	2.04E-03
SMU_1165c	acrR, Transcription				2.656	2.38E-03
SMU_1166c	psaB, Transport and binding	-2.456	6.07E-04			
SMU_1175	dagA, Transport and binding				2.419	1.80E-04
SMU_1177c	atmA, Transport and binding				1.942	2.95E-03
SMU_1178c	Transport and binding				2.246	5.23E-04
SMU_1179c	Transport and binding				1.998	2.48E-03
SMU_1180	phnA, Central Metabolism				2.749	3.37E-05
SMU_1187	glmS, Cell division/envelope	2.387	6.57E-05		1.902	6.57E-05
SMU_1188	sipC, Protein fate				-1.916	2.40E-03
SMU_1211	Central metabolism				2.710	3.38E-05
SMU_1217c	atmA, Transport and binding	-2.169	2.29E-03		-2.706	2.29E-03
SMU_1235	thdF, Biosynthesis: Macromolecules and co-factors				-2.601	2.75E-05
SMU_1243	ltrA, Stress tolerance/detoxification	-2.003	3.40E-03		-2.250	3.40E-03
SMU_1249c	Hypothetical	2.176	9.36E-04		2.214	9.36E-04
SMU_1250c	Hypothetical	2.344	7.42E-04		2.957	7.42E-04
SMU_1257c	Hypothetical				-2.364	9.05E-05
SMU_1260c	Hypothetical	-2.388	1.20E-03		-2.393	1.20E-03
SMU_1261c	hisE, Biosynthesis:				-3.175	1.84E-03

	Macromolecules and co-factors				
SMU_1262c	Hypothetical	-2.181	8.97E-04	-2.505	8.97E-04
SMU_1263	hisI hisE, Biosynthesis: Macromolecules and co-factors			-2.885	4.48E-03
SMU_1264	hisF, Biosynthesis: Macromolecules and co-factors	-2.791	3.48E-04	-2.945	3.48E-04
SMU_1265	hisA, Biosynthesis: Macromolecules and co-factors	-2.939	5.36E-06	-3.278	5.36E-06
SMU_1266	hisH, Biosynthesis: Macromolecules and co-factors			-2.967	2.38E-03
SMU_1267c	Hypothetical	-2.937	9.96E-05	-3.396	9.96E-05
SMU_1270	hisD, Biosynthesis: Macromolecules and co-factors	-2.812	1.96E-03	-2.954	1.96E-03
SMU_1272	hisZ, Biosynthesis: Macromolecules and co-factors	-3.062	2.86E-04	-3.010	2.86E-04
SMU_1273	hisC, Biosynthesis: Macromolecules and co-factors	-2.623	1.59E-03	-3.151	1.59E-03
SMU_1293c	Hypothetical			-2.568	1.10E-05
SMU_1295	add, Biosynthesis: Macromolecules and co-factors			-3.176	1.06E-05
SMU_1296	yghU, Hypothetical	-5.304	3.78E-11	-7.022	3.78E-11
SMU_1297	Hypothetical	-8.633	0	-10.282	0
SMU_1299c	Hypothetical	2.698	5.46E-04	2.967	5.46E-04
SMU_1301c	Hypothetical	2.634	5.78E-05		
SMU_1308	aldR, Biosynthesis: Macromolecules and co-factors			2.075	3.60E-03
SMU_1309c	gldA-2, Energy metabolism			2.669	1.07E-05
SMU_1315c	Transport and binding	2.326	5.18E-04	3.206	5.18E-04
SMU_1316c	Hypothetical	2.077	3.22E-03	3.400	3.22E-03
SMU_1317c	Hypothetical	2.962	2.36E-04	4.399	2.36E-04
SMU_1321c	Hypothetical			2.121	1.41E-03
SMU_1323	Hypothetical			-3.274	3.41E-06
SMU_1327c	Transport and binding			2.324	7.93E-04
SMU_1349	Hypothetical	2.318	7.13E-04	4.168	7.13E-04
SMU_1379	tpn, DNA metabolism			-2.282	5.43E-04
SMU_1393c	Hypothetical	-2.094	3.84E-03		
SMU_1394	lepA, Other	-2.090	5.21E-04		

SMU_1396	gbpC, Pathogenesis			-10.780	0
SMU_1400c	Hypothetical	2.853	1.06E-04	4.070	1.06E-04
SMU_1411	Hypothetical			-3.113	3.84E-03
SMU_1412c	yhcA, Transport and binding			-3.695	3.23E-07
SMU_1416c	mutT, DNA metabolism			2.122	1.66E-03
SMU_1418	hemN, Redox homeostasis			2.401	8.95E-05
SMU_1425	clpB, Protein fate	-2.480	8.08E-04		
SMU_1438c	prtB, Protein fate			-2.888	1.63E-05
SMU_1447c	Transport and binding			1.975	0.003110<br>2
SMU_1451	aldB, Other	-3.520	1.20E-08	-4.883	1.20E-08
SMU_1452	alsS, Energy metabolism	-3.639	6.47E-10	-4.681	6.47E-10
SMU_1461	rfbA rmlA, Cell division/envelope			2.215	03.32E-<br>04
SMU_1479	Hypothetical			-4.517	2.63E-07
SMU_1502c	Hypothetical			2.2056	3.63E-03
SMU_1509	rggD, Transcription	2.072	4.47E-03		
SMU_1513	smc, Cell division/envelope			-1.956	1.52E-03
SMU_1519	glnQ, Transport and binding	2.465	1.41E-03		
SMU_1523	endA, Competence	-2.226	6.05E-04	-4.489	6.04E-04
SMU_1524c	epuA, Hypothetical	-2.178	3.08E-03	-3.115	3.08E-03
SMU_1525	murA, Cell division/envelope			-2.943	2.03E-07
SMU_1533	atpB, Energy metabolism			1.843	3.95E-03
SMU_1541	pul, Energy metabolism			-1.967	8.54E-04
SMU_1543	ligA, DNA metabolism			-1.938	1.43E-03
SMU_1545c	Hypothetical	2.623	1.42E-04	4.074	1.42E-04
SMU_1546	Hypothetical			2.219	2.57E-03
SMU_1561	trkB , Transport and binding	-2.538	3.48E-05	-4.751	3.48E-05
SMU_1562	trkA, Transport and binding	-2.499	2.54E-05	-5.643	2.54E-05
SMU_1563	pacL, Transport and binding	-2.708	9.61E-07	-5.068	9.61E-07
SMU_1565	malM, Energy metabolism	2.472	1.10E-03	2.5414	1.10E-03
SMU_1566	malR, Transcription	-4.121	6.34E-11	-2.290	6.34E-11
SMU_1568	malE, Transport and binding	3.203	7.48E-07	2.555	7.48E-07
SMU_1572	murZ, Cell division/envelope	-2.048	4.43E-04		
SMU_1574c	Hypothetical			-1.982	1.64E-03
SMU_1590	amy, Energy metabolism			-2.198	3.73E-04
SMU_1592	pepQ, Protein fate	-2.195	6.13E-04		

SMU_1593c	Hypothetical	-2.745	8.16E-06			-3.262	8.16E-06
SMU_1604c	Hypothetical	2.582	2.62E-04			2.649	2.62E-04
SMU_1616c	Hypothetical					-2.100	3.45E-03
SMU_1629c	Cell division/envelope					-1.807	4.03E-03
SMU_1641c	Hypothetical	-1.957	9.98E-04			-1.961	9.98E-04
SMU_1644c	Hypothetical	2.153	4.14E-04				
SMU_1645	tehB, Pathogenesis	-4.862	2.28E-13			-9.493	2.28E-13
SMU_1648c	Hypothetical					-2.389	2.37E-03
SMU_1649	exoA smxA, Biosynthesis: Macromolecules and co-factors	-4.727	3.87E-12			-12.358	3.87E-12
SMU_1650	end3, DNA metabolism	-3.618	1.40E-06			-5.789	1.40E-06
SMU_1653	serA, Biosynthesis: Macromolecules and co-factors					1.991	1.35E-03
SMU_1654c	Hypothetical	1.980	1.91E-03			2.622	1.91E-03
SMU_1655c	Hypothetical					3.018	1.65E-04
SMU_1656	serC, Biosynthesis: Macromolecules and co-factors	2.031	1.54E-03			2.657	1.54E-03
SMU_1664c	acoB, Biosynthesis: Macromolecules and co-factors					-2.064	4.40E-04
SMU_1667	livM, Transport and binding	-2.701	3.83E-03				
SMU_1668	livH, Transport and binding	-2.716	1.41E-06			-2.496	1.41E-06
SMU_1669	livK, Transport and binding	-2.338	5.16E-04			-2.175	5.20E-04
SMU_1671c	Hypothetical					-2.510	9.96E-05
SMU_1674	patB, Central Metabolism					-2.711	3.47E-05
SMU_1675	metB, Biosynthesis: Macromolecules and co-factors					-2.601	1.63E-04
SMU_1679c	Hypothetical					-4.476	2.59E-09
SMU_1680c	Hypothetical					-4.065	8.15E-09
SMU_1681c	Hypothetical					-3.717	4.17E-08
SMU_1685c	Hypothetical	2.111	2.60E-03			2.002	2.60E-03
SMU_1692	pflA pflC, Other	-11.230	0			-13.979	0
SMU_1702c	Signal transduction	-4.285	4.90E-04				
SMU_1703c	Hypothetical	-4.288	1.52E-07			-3.300	1.52E-07
SMU_1719c	Hypothetical					-2.079	8.62E-04
SMU_1722c	Hypothetical					-3.028	3.56E-07
SMU_1723c	Hypothetical					-2.864	4.50E-07
SMU_1727	Cell division/envelope			3.544	7.19E-06	3.935	7.19E-06

SMU_1746c	fabM, Biosynthesis: Macromolecules and co-factors			-2.290	4.47E-05
SMU_1752c	Hypothetical	4.944	1.69E-11		
SMU_1753c	Hypothetical	2.269	6.85E-04		
SMU_1754c	Hypothetical	2.091	2.39E-03		
SMU_1760c	Hypothetical	2.108	1.21E-03		
SMU_1764c	Hypothetical	2.118	1.08E-03		
SMU_1787c	Protein fate	-3.956	2.24E-10	-2.809	2.24E-10
SMU_1788c	bta, Transport and binding	-11.150	0	-10.941	0
SMU_1812	tpn, DNA metabolism	-2.956	3.34E-06	-2.036	3.34E-06
SMU_1813	DNA metabolism	-9.123	6.59E-04		
SMU_1814	scnK, Signal transduction	-7.166	0	-6.391	0
SMU_1815	scnR, Signal transduction	-7.023	0	-7.813	0
SMU_1822	aspS, Biosynthesis: Macromolecules and co-factors			-1.926	2.63E-03
SMU_1848	Hypothetical			-3.098	1.50E-06
SMU_1849	comEB, Competence	-2.394	7.58E-05	-5.949	7.58E-05
SMU_1850	pepP, Protein fate			-5.564	1.89E-04
SMU_1851	uvrA, DNA metabolism	-2.401	6.27E-04	-6.369	6.27E-04
SMU_1854	hdrR, Pathogenesis			3.564	2.38E-03
SMU_1861c	Hypothetical			5.046	1.54E-09
SMU_1865	mutY, DNA metabolism	-5.807	2.06E-13	-9.585	2.06E-13
SMU_1867c	adhB, Energy metabolism			-21.509	0
SMU_1869	trxA, Stress tolerance/detoxification			-23.483	0
SMU_1876	Hypothetical			3.305	7.35E-07
SMU_1877	manL, Signal transduction			1.870	4.01E-03
SMU_1879	manN, Signal tranduction			1.890	2.96E-03
SMU_1882c	Hypothetical			2.450	3.50E-05
SMU_1889c	Hypothetical			2.293	3.57E-03
SMU_1895c	Hypothetical			5.802	1.01E-12
SMU_1896c	Hypothetical			3.304	1.92E-07
SMU_1897	Transport and binding			3.157	1.86E-05
SMU_1902c	Hypothetical	3.839	1.00E-05		
SMU_1904c	Hypothetical	-2.210	1.43E-03		
SMU_1906c	Hypothetical	-2.508	5.65E-04		
SMU_1907	Hypothetical	-2.988	6.80E-05		

SMU_1909c	Hypothetical	-3.296	2.63E-06		
SMU_1910c	Hypothetical	-3.122	7.11E-06		
SMU_1912c	Hypothetical	-2.998	4.34E-03		
SMU_1913c	Other	-3.566	2.19E-07	-2.182	2.19E-07
SMU_1914c	bip, Pathogenesis	-3.273	1.51E-06		
SMU_1915	comC, Competence	-8.508	1.95E-05	-9.922	1.95E-05
SMU_1916	comD, Signal transduction	-8.216	1.44E-14	-7.125	1.44E-14
SMU_1917	comE, Signal transduction	-10.643	5.03E-12	-8.569	5.03E-12
SMU_1919	sapR, Hypothetical			-1.986	1.55E-03
SMU_1920	pdgA, Other			-2.124	2.25E-04
SMU_1921	dnaI, DNA metabolism			-1.951	1.56E-03
SMU_1924	gcrR, Signal transduction	1.883	3.08E-03		
SMU_1929	htpX, Stress tolerance/detoxification			-2.515	1.31E-05
SMU_1930	lemA, Other			-2.119	4.34E-04
SMU_1931	gidB, Cell division/envelope			2.038	2.66E-03
SMU_1949	pbp2a, Cell division/envelope			-2.069	1.18E-03
SMU_1954	groEL, Protein fate			-3.735	5.40E-09
SMU_1955	groES, Protein fate			-3.627	1.15E-09
SMU_1961c	levD, Signal tranduction			2.169	4.42E-04
SMU_1973	Protein fate			2.267	3.42E-04
SMU_1974	proC, Biosynthesis: Macromolecules and co-factors	2.093	2.24E-03		
SMU_1975c	Hypothetical	2.901	8.45E-06	3.018	8.45E-06
SMU_1976c	Hypothetical	3.123	1.89E-04	2.519	1.89E-04
SMU_1977c	Hypothetical	3.171	2.93E-04		
SMU_1987	comYA, Competence	-2.557	1.68E-03		
SMU_1988c	Hypothetical	-11.124	0	-30.255	0
SMU_1992	tyrS, Biosynthesis: Macromolecules and co-factors	2.007	1.53E-03	2.544	1.53E-03
SMU_1994	adcC, Transport and binding			-2.921	1.10E-04
SMU_1995c	adcR, Transcription			-2.423	2.72E-03
SMU_2027	Transcription			-2.623	4.32E-05
SMU_2028	ftf, Energy metabolism			2.337	6.67E-05
SMU_2029	clpC, Protein fate			-2.404	6.92E-05
SMU_2036	pepO, Protein fate			-6.736	0
SMU_2037	dexS, Energy metabolism			2.515	2.25E-04

SMU_2038	treB, Signal transduction					2.993	1.22E-05
SMU_2042	dexT, Energy metabolism	-1.823	4.34E-03				
SMU_2043c	Dtd, Biosynthesis: Macromolecules and co-factors	-3.598	1.68E-06			-3.267	1.68E-06
SMU_2044	relA, Stress tolerance/detoxification	-3.414	3.08E-09			-3.594	3.08E-09
SMU_2047	ptsG, Transport and binding	1.931	2.28E-03			2.337	2.28E-03
SMU_2056	cshA, Cell division/envelope					-2.245	9.50E-04
SMU_2059c	Hypothetical					2.519	1.93E-04
SMU_2064c	Hypothetical					2.134	4.50E-04
SMU_2074	nrdD, Biosynthesis: Macromolecules and co-factors					2.562	1.36E-05
SMU_2077c	Hypothetical					-2.491	2.73E-05
SMU_2078c	DNA metabolism					-2.319	3.70E-03
SMU_2081	Hypothetical			-3.142	1.00E-03		
SMU_2083c	Hypothetical			-3.563	1.66E-04	-1.905	1.66E-04
SMU_2084c	spxB, Transcription			-1385.509	8.22E-14	-452.248	8.22E-14
SMU_2085	recA, DNA metabolism	-1.961	1.46E-03			-2.304	1.46E-03
SMU_2091c	hexA, DNA metabolism					-2.209	1.30E-03
SMU_2102	hisS, Biosynthesis: Macromolecules and co-factors	-3.110	3.90E-08			-3.287	3.90E-08
SMU_2112	gbpA, Pathogenesis					2.616	6.27E-06
SMU_2127	gabD Central metabolism					-2.327	2.39E-04
SMU_2130	shetA, Stress tolerance/detoxification			2.850	4.08E-03		
SMU_2133c	Hypothetical			3.666	4.78E-05	2.131	4.78E-05
SMU_2142	rpiA, Energy metabolism	-1.982	2.25E-03			-2.032	2.25E-03
SMU_2143c	trmU, Biosynthesis: Macromolecules and co-factors	-2.102	2.71E-04			-2.145	2.71E-04
SMU_2153c	Protein fate					-5.372	4.22E-11
SMU_2154c	Protein fate					-5.594	1.74E-11
SMU_2155	Hypothetical					-2.114	2.96E-03
SMU_2156	recF, DNA metabolism					-2.435	7.54E-05
SMU_2157	guaB, Biosynthesis: Macromolecules and co-factors	1.880	3.24E-03			2.554	3.23E-03

Table S3. qRT-PCR validation of gene expression trends in H_2O_2-exposed *S. mutans* UA159 as compared to unstressed controls.

		UA159 H_2O_2 vs. UA159 control			
		RNA-Seq		qRT-PCR	
**Gene**	**Description**	**Fold change**	***p*-value**	**Fold change**	***p*-value**
SMU_0131	lplA, lipoate-protein ligase	6.913	6.53E-14	4.307	0.0123
SMU_0137	mleS, Energy metabolism	ND[a]	ND	1.342	0.6487
SMU_0995	yclN, Transport and binding	ND	ND	0.549	0.4589
SMU_1273	hisC, histidine biosynthesis	5.038	2.72E-04	6.160	0.0726
SMU_1452	alsS, Energy metabolism	ND	ND	1.895	0.1901
SMU_1692	pflA, pyruvate formate lyase	3.553	1.08E-06	7.150	0.0245
SMU_1916	comD, Competence	4.394	5.89E-06	5.420	0.0379

[a] ND, no significant difference in gene expression was determined.

Table S4. qRT-PCR validation of gene expression trends in H_2O_2-exposed *S. mutans* ∆*spxA1* or ∆*spxA1*/∆*spxA2* as compared to H_2O_2-exposed UA159.

		∆*spxA1* H_2O_2 vs. UA159 H_2O_2				∆*spxA1*/∆*spxA2* H_2O_2 vs. UA159 H_2O_2			
		RNA-Seq		qRT-PCR		RNA-Seq		qRT-PCR	
**Gene**	**Description**	**Fold change**	***p*-value**	**Fold change**	***p*-value**	**Fold change**	***p*-value**	**Fold change**	***p*-value**
SMU_0131	lplA, lipoate-protein ligase	-10.559	0	-16.167	0.0022	-16.015	0	-12.170	0.0025
SMU_0137	mleS, Energy metabolism	-5.095	3.91E-10	-5.708	0.0410	-7.283	3.91E-10	-5.669	0.0417
SMU_0995	yclN, Transport and binding	14.552	7.03E-10	185.410	0.0044	15.896	7.03E-10	591.342	0.0201
SMU_1273	hisC, histidine biosynthesis	-2.623	1.59E-03	-3.275	0.1121	-3.151	1.59E-03	-3.275	0.1135
SMU_1452	alsS, Energy metabolism	-3.639	6.47E-10	-2.872	0.0243	-4.681	6.47E-10	-2.56	0.0339
SMU_1692	pflA, pyruvate formate lyase	-11.230	0	-22.074	0.0161	-13.979	0	-42.491	0.0146
SMU_1916	comD, Competence	-8.216	1.44E-14	-15.072	0.0212	-7.125	1.44E-14	-9.649	0.0241

Table S5. Primers used for gene inactivation.

Primer	Sequence	Application
gdhADel1	5'-CGCCGGTGGTCAAGACATGAAG-3'	*gdhA* gene deletion
gdhADel2	5'-GGATTGGGATTATACAGGCG-3'	
gdhADel3	5'-CATGTAATGATAAGCACAGATATACGCCCTATTGT AGTTTTCCTAGACTC-3'	
gdhADel4	5'- GCGTATATCTGTGCTTATCATTACATGCTTAAAGAGCTT AAAGAAGTACAGC-3'	
gdhADel5	5'-GGTCCGGACAGATGTTCAACACC-3'	
gdhADel6	5'-GCTGTAGTGAAAGTGCTCAAACC-3'	
5'gdhADelScreen	5'-CCGGTAGGTGAGGTAGG-3'	
3'gdhADelScreen	5'-GGACAATCGGATACCTTGC-3'	
lplADel1	5'-GAAGGTGATGAGGTCAAAGAAGGGGAG-3'	*lplA* gene deletion
lplADel2	5'-CAAGCAGGTGAAGTTCTTGAAATAG-3'	
lplADel3	5'- CATGTAATGATAAGCAATAGATATAGCCGACTGATAAT A TATTTCATTTTCTCTCC-3'	
lplADel4	5'-GGCTATATCTATTGCTTATCATTACATG TAGGCCTAGGTTATG-3'	
lplADel5	5'-GCTGGCAAAGTAAGCGTAGAGTG-3'	
lplADel6	5'-GCAACTTGAAGTATCTTGTTGCGC-3'	
5'lplADelScreen	5’-CCTTCTCGGAGGTTATGTATG-3’	
3'lplADelScreen	5’-GGATGTTGATGAAGATAATGCCG-3’	
hisCDel1	5'-CTGACGAAAGTCCAACTCCCCTCG-3'	*hisC* gene deletion
hisCDel2	5'-GGTAATATCGGCAACTTGCTG-3'	
hisCDel3	5'- CATGTAATGATAAGCAATAGATATAGCCCCATAAACAC AGAAGACTATTTAG-3'	
hisCDel4	5'- GGCTATATCTATTGCTTATCATTACATGGCACTCAAGA ATGTGTTATC-3'	
hisCDel5	5'-CGTGGAGGGAGATTCAGCTGG-3'	
hisCDel6	5'-CCGTAACCGTGAATTTCAAGC-3'	
5'hisCDelScreen	5'-GCTTGAAGAGCTTATCGTGC-3'	
3'hisCDelScreen	5'-GCTCTTGTGGCCAATATCAAAGG-3'	
adhDDel1	5'-GACTCGGCAACTAATGAAGGCTC-3'	*ahdD* gene deletion
adhDDel2	5'-CCATGAGTCTGTTAATTTGGCAGCTG-3'	
adhDDel3	5'-CATGTAATGATAAGCAATAGATATAGCC CATAATAATTTCGACTGCCATTTTCTTCAC-3'	
adhDDel4	5'-GGCTATATCTATTGCTTATCATTACATG CGCCAAAGCGTCGTTAATG-3'	
adhDDel5	5'-GAAGTCTGGTTATTAACCAATTGGGCCC-3'	
adhDDel6	5'-AATTGGGCCCAAGATTGCTGG-3'	
5'adhDDelScreen	5’-GGTGTAGGAGCAACTATCC-3’	
3'adhDDelScreen	5’-AGCTTGGGACTTGCC-3’	
5'alsSDel	5'-GCCCTCAATCAGGCCAGCCTG-3'	*alsS* gene deletion
5'alsSBamHI	5'-GCGGGATCCATTTCGGTCATAAAAAGTCC-3'	
3'alsSBamHI	5'-GCGGGATCCCCTGATGAATTCTACTAAAAAGGAG-3'	
3'alsSDel	5'-GCCAACATGGGAAGGCGACTATG-3'	
5'alsSDelScreen	5’-GACACAAAAGCTCTTGAG-3’	
3'alsSDelScreen	5’-CACCCACAGTTAAAGAACC-3’	

Table S6. Primers used for qRT-PCR.

Primer	Sequence
lplARTFwd	5'-GCTCATGACATTCATGTCGTTCGTCG-3'
lplARTRev	5'-GCCATCAATTTCAAGATCATTGCGTCC-3'
mleSRTFwd	5'-AGAGCAAGCAGAGCAGAC-3'
mleSRTRev	5'-AGATCACTGTAACCTTCAATCG-3'
smu995RTFwd	5'-GGATGATGTTTTCATTCCTCTAGTAGG-3'
smu995RTRev	5'-GCCAGCTATAGTAAACTGATGAGC-3'
hisCRTFwd	5'-GGTGCTAGTCCCAAAGTTCG-3'
hisCRTRev	5'-GCCAAGAAAGCGATAGATAAAACATCATCTG-3'
alsSRTFwd	5'-CCTGGTGTTGTGATTACAACCAGC-3'
alsSRTRev	5'-GCTTTAGCCAAACGATAGGCATTAGC-3'
pflARTFwd	5'CCTGGTCTGACTGATCGGGATG-3'
pflARTRev	5'-TGTTGGCGGTTTAACACCTTCC-3'
comDRTFwd	5'-TTGCTGTTACGATGGTGAACG-3'
comDRTRev	5'-TGCTACTGCCCATTACAATTCC-3'
